# A systematic review of machine learning in heart disease prediction

**DOI:** 10.55730/1300-0152.2766

**Published:** 2025-09-11

**Authors:** Tathagat BANERJEE, İshak PAÇAL

**Affiliations:** 1 Department of Computer Engineering, Indian Institute of Technology, Patna, Bihar, India; 2 Department of Computer Engineering, Faculty of Engineering, Igdir University, Iğdır, Turkiye; 3 Department of Electronics and Information Technologies, Faculty of Architecture and Engineering, Nakhchivan State University, Nakhchivan, Azerbaijan

**Keywords:** Heart disease, machine learning, deep learning, predictive modeling, explainable artificial intelligence

## Abstract

**Background/aim:**

Cardiovascular diseases (CVDs) are a leading cause of global mortality, prompting the need for advanced predictive tools. While machine learning (ML) offers a powerful solution, there are significant challenges to clinical translation. This systematic review synthesizes the current state of ML in heart disease prediction, evaluating algorithmic performance, data utilization, and key translational challenges.

**Materials and methods:**

Following PRISMA guidelines, a systematic search of literature published up to 2025 was conducted. From an initial pool of over 2500 records, a rigorous screening process yielded 65 studies for in-depth qualitative synthesis.

**Results:**

Analysis showed that ensemble learning models dominate prediction tasks on structured data, achieving high accuracy on benchmarks. Deep learning (DL) is increasingly applied to unstructured data like electrocardiogram signals and cardiac imaging. Despite high performance reported in models, a significant translational gap exists. This is driven by a pervasive lack of external validation, an overreliance on limited public datasets, and the black-box nature of complex models that reduces clinical trust. The adoption of explainable artificial intelligence is a key trend aimed at mitigating these challenges.

**Conclusion:**

While ML shows significant potential, its utility remains largely confined to academic settings. The future of the field depends on a fundamental research shift, rather than on incremental accuracy gains. Progress requires a concerted focus on robust external validation, the development of large-scale representative datasets, and the creation of interpretable systems that can be effectively integrated into clinical workflows to improve patient outcomes.

## 1. Introduction

The heart maintains life by pumping blood through a complex set of blood vessels, keeping the human body alive. Blood circulation nourishes all body tissues with oxygen and other important nutrients, while expelling air and other metabolic waste (Lindstrom et al., 2025). Reduced heart efficiency directly reduces the functional capability of all other organs, such as the brain, kidneys, and lungs, highlighting the crucial role the heart plays in maintaining the human body and homeostasis. The heart pumps continuously by rhythmic contractions that are governed by intrinsic electrical impulses and reinforced by a synchronized arrangement of 4 chambers and valves (Aghapanah et al., 2024).

Furthermore, the heart plays a key role in regulating body temperature, maintaining blood pressure, and balancing hormones and biochemicals in the blood (Rwebembera et al., 2021). However, as the heart is central to all activities, it is prone to congenital and acquired diseases. Congenital heart defects, cardiomyopathies, and age-related degenerative changes are naturally occurring cardiovascular conditions (Pirillo and Norata, 2023). Diseases associated with an unhealthy lifestyle, including coronary artery disease (CAD), myocardial infarction (heart attack), hypertensive heart disease, and heart failure, are frequently correlated with poor eating habits, lack of physical exercise, smoking, excessive alcohol abuse, obesity, and chronic stress (Rani et al., 2024a).

Cardiovascular diseases (CVDs) are the primary cause of death globally. The World Health Organization (WHO) estimates about 17.9 million people die annually due to CVDs, and this accounts for almost 32% of all deaths in the world. More than 75% of CVD-related deaths are registered in low- and middle-income countries, highlighting the lack of access to early detection and treatment (Ma et al., 2023). In addition, most of these disorders progress gradually and may not be symptomatic until the advanced stages, meaning early detection and treatment are therefore essential (Ma and Huang, 2018). Improving health outcomes demands an approach to address preventable heart disease and the inherent risks that cannot be prevented.

Machine learning (ML) approaches have become powerful tools to interpret complicated clinical data, facilitating the early prediction and fragmentation of cardiac events (Rani et al., 2024a). These strategies assist clinicians in plotting patterns using high-dimensional data on electrocardiograms (ECGs), echocardiography, angiograms, cardiac MRI, and electronic health records (EHRs) (Aghapanah et al., 2024). ML can analyze parameters such as left ventricular ejection fraction, QT interval, ST elevation, heart rate variability, and troponin levels to identify irregularities originating within acute coronary syndromes, atrial fibrillation, congestive heart failure, and ischemic heart diseases (Das et al., 2023). Moreover, diabetes mellitus, hypertension, hyperlipidaemia, obesity, and chronic inflammation are usually modeled as risk factors in supervised learning models (Mehdizadeh et al., 2022).

The complications of heart disease include ventricular hypertrophy, pulmonary edema, stroke, and renal impairment, warranting the creation of sound prognostic systems (Rani et al., 2024b). As the heterogeneity and amount of medical data increases, combining ML with clinical knowledge can provide a gateway to precision medicine (Ma et al., 2024). Support vector machine (SVM), random forest (RF), gradient boosting machine (GBM), and deep neural networks (DNNs) have been successfully used to stratify patients into risk categories and predicting the probability of developing complications (Das et al., 2023). A previous systematic review of ML in predicting heart-related diseases emphasized the type of heart-related conditions being predicted, the medical variables applied, and the clinical applicability of the results in the prediction (Rani et al., 2024b). Moreover, Rani et al. (2024b) states that these models deal with the effects of imbalanced data, provide no explanations, and lack generalizability in different populations. The goal of the current review is to highlight the usefulness of these models in cardiology (Hoffman and Kaplan, 2002).

### 1.1. The global impact of cardiovascular disease

CVD, which constitutes a complex of disorders, including CAD, heart failure, arrhythmias, and congenital heart defects, is the leading cause of morbidity and mortality globally (Babu et al., 2024). Over 75% of CVD-related deaths were reported in low- and middle-income countries, emphasizing the growing necessity of high-quality, affordable, and accurate diagnostic and predictive tools.

The global increase in heart disease is of great concern to global public health and the economy (Sreeja et al., 2024). The increasing burden on healthcare systems to treat chronic diseases, prevent acute cardiac events, and enhance patient outcomes has resulted in a dissatisfactory state of care (Griffeth et al., 2024). Although effective, conventional diagnostic systems, including angiography, echocardiography, and ECG-based assessments, have limitations such as high expenditure, invasiveness, operator dependency, and lack of accessibility in distant places (Choi et al., 2020).

Furthermore, many people do not get a diagnosis until the onset of a severe condition like myocardial infarction or stroke. Therefore, it is essential to detect the risk of heart disease early and accurately based on available clinical and lifestyle information. The development of computational medicine and artificial intelligence (AI), especially ML, is now being used to address this problem by providing predictive, scalable, and explainable solutions that could be implemented in existing clinical practices (Ma et al., 2024; Stonier et al., 2024).

### 1.2. The importance of predictive modeling in cardiology

Prior cohort risk analysis and early prediction can be used to enhance cardiovascular outcomes (Guarneros-Nolasco et al., 2021). Predictive modeling in cardiology can promote actions and measures to prevent the development of clinical symptoms by allowing clinicians to recognize high-risk individuals before the onset of clinical signs and symptoms. Linear models (Nakamori et al., 2024) and traditional logistic regression (LR)-based risk scores, which include the Framingham risk score (FRS) and SCORE models, have been validated but are limited by linear assumptions and only a few variables (Yasuhara and Garg, 2021).

The paradigm shift in predictive models comes with ML that can model complex and nonlinear relationships among heterogeneous features (Kwan et al., 2024). ML-based models can potentially consume vast amounts of patient data, such as clinical history, laboratory measurements, ECG traces, imaging data, and demographic characteristics, to derive patterns and relationships that would otherwise remain unidentifiable through traditional statistical analysis (Guarneros-Nolasco et al., 2021).

The potential of ML is emphasized in a few recent studies. Al-Alshaikh et al. (2024) compared the performance of various ML algorithms in predicting heart diseases, stressing the advantage of ensemble learning and the use of explainability to ensure clinical interpretability. Similarly, Rehman et al. (2025) used an ML-based classifier to better predict cardiovascular risk. Advanced classification models like extreme gradient boosting (XGBoost) and LightGBM have outperformed LR and decision trees for heart disease prediction (Ansari et al., 2023).

Predictive modeling may also help in prognosis, treatment plans, and patient response follow-ups (Mehdizadeh et al., 2022). For example, Griffeth et al. (2024) used ML models to predict the probability of reoperation in adults with congenital heart disease. This highlights the effectiveness of predictive analytical techniques concerning long-term care and surgery program optimization.

### 1.3. Overview of ML paradigms

ML is an area of AI that creates algorithms capable of absorbing patterns in the data and issuing informed predictions without being specifically coded (Al-Alshaikh et al., 2024; Pacal, 2025). Compared with conventional statistical tools, ML algorithms can model complex and nonlinear associations in broad and heterogeneous data sets (Ince et al., 2025; Pacal et al., 2025). These approaches have become popular in the healthcare domain since they have the potential to address multifactorial inputs, noise tolerance, and adaptation to fluctuating clinical processes (Rimal et al., 2024).

ML can be used in several areas in cardiology, such as early disease identification, prediction, risk, and optimizing treatment. For example, ML models have shown superior performance to traditional LR-based tools, including the FRS, in predicting unfavorable cardiovascular outcomes due to their capacity to interpret nonlinear relationships and the interaction among variables (Rehman et al., 2025). Al-Alshaikh et al. (2024) showed that ensemble ML models surpass the classification performance of single classifiers regarding heart disease prediction. Furthermore, explainable AI (XAI) techniques, such as Shapley additive explanations (SHAP) and local interpretable model-agnostic explanations (LIME), have been integrated as used as clinical transparency tools (Mesquita and Marques, 2024; Sreeja et al., 2024; Talukder et al., 2025).

Though many ML algorithms can predict heart disease, they are all different in their working mechanism and capabilities. Such models are widely used as decision trees, SVMs, artificial neural networks (ANNs), and ensemble methods (e.g., RF and GBM) (Zhu et al., 2023; Duan et al., 2024; Islam et al., 2024; Pingitore et al., 2024; Rani et al., 2024b; Saheed et al., 2024; Bhavekar et al., 2024; El-Sofany et al., 2024).

XGBoost is a very effective ensemble learning algorithm. XGBoost is an extension of gradient-boosting decision trees, with the addition of regularization, parallel tree training, and handling of missing data. It has many applications in the medical field because of its interpretability, accuracy, and scalability. According to Rehman et al. (2025), XGBoost is considerably better than traditional classifiers, LR, and decision trees, when used in cardiovascular risk stratification. This model handled imbalanced data exceptionally well, a common problem in clinical research. Another noteworthy algorithm is LightGBM, which offers faster training and lower memory usage than XGBoost while maintaining comparable accuracy (Gopi et al., 2024). Combined with XAI techniques, these advanced algorithms help bridge the gap between black-box models and clinical trust (Sreeja et al., 2024; Mesquita and Marques, 2024; El-Sofany et al., 2024; Talukder et al., 2025).

Medical diagnostics and predictive analytics are the most promising fields of ML, especially in cardiology. ML models provide an opportunity to detect individuals at risk as early as possible, better stratify patients, and personalize treatment plans. Medical imaging, ECG, blood biomarkers, and demographics can all be analyzed with ML to pick up the subtle patterns, which often cannot be discovered with traditional statistical analysis (Al-Alshaikh et al., 2024; Griffeth et al., 2024).

Recent studies have provided comprehensive overviews of ML and DL frameworks applied to heart disease classification, emphasizing advancements in predictive accuracy and clinical integration (Rani et al., 2024a; Karna et al., 2025a). These reviews underscore the potential of deep neural architectures in interpreting complex cardiac imaging and physiological datasets for more reliable diagnostic support. Supervised, unsupervised, semisupervised, and reinforcement learning are broad categories of ML. Supervised learning is the most common for heart disease prediction because labeled medical data is available for model training.

Supervised learning algorithms are trained on input–output examples, and are often used in category prediction (e.g., classifying patients to risk categories) or numerical prediction (e.g., predicting degrees of risk). SVM, RF, k-nearest neighbors (k-NN), LR, GBM, and DL models, including convolutional neural networks (CNNs) and recurrent neural networks (RNNs), have also been considered in cardiovascular studies. ML, especially DL architectures, have become instrumental in biomedical classification and regression tasks, significantly improving diagnostic accuracy and clinical decision-making.

Regarding pattern discovery, patient segmentation, and outlier detection, unsupervised learning techniques are utilized through clustering (e.g., k-means and density-based spatial clustering of applications with noise (DBSCAN)) and dimensionality reduction (e.g., principal components analysis (PCA) and t-distributed stochastic neighbor embedding (t-SNE)). As a specific example, various feature fusion strategies were considered to merge features of tongue images to classify heart diseases in a noninvasive fashion by prior dimension reduction using unsupervised methods, followed by the classification process (Duan et al., 2024).

DL is a subset of ML capable of extracting abstract hierarchical features from raw data in an automated fashion, making it a popular choice in imaging and signal processing applications. Pachiyannan et al. (2024) proposed a DL-based method that processed ECG readings to detect the presence of congenital heart disease at early stages of the disease. Rimal and Sharma (2024) comparatively evaluated the performance of automated ML (AutoML) and ensemble approaches such as AdaBoost. In all experiments, both ensemble approaches (like AdaBoost) and AutoML techniques outperformed traditional single learners on various datasets

### 1.4. DL for cardiac imaging

DL has emerged as a transformative tool in medical image processing, with remarkable success across a range of applications. DL is paving the way for significant advancements in diagnostics and patient care (Lubbad et al., 2024; Pacal and Attallah, 2025a). DL models, particularly CNNs, can automatically learn intricate patterns and features from vast datasets of medical images, often exceeding human-level accuracy in tasks such as classification, segmentation, and anomaly detection (Aruk et al., 2025; Pacal and Attallah, 2025b). In this context, the impact on cardiac imaging has been particularly profound. DL has currently been integrated into cardiac imaging and has yielded significant results in cardiovascular pathology assimilation and diagnosis. Such DL models have become a core part of modern cardiac diagnostics, ranging from improving the segmentation of the images to disease-specific phenotyping. Using cardiac CT, MRI, SPECT, PET, and multimodal strategies, researchers have examined structural, functional, and molecular manifestations of heart diseases, especially ventricular pathology, cardiomyopathy, and valvular disease. This integration not only accelerates and refines the diagnostic process but also enables a more detailed and quantitative understanding of cardiac conditions, ultimately leading to more personalized and effective treatment strategies.

One of the DL applications in cardiac imaging is automatic segmentation of cardiac structures. Sun et al. (2024) showcased the successful performance of AI in segmenting left ventricular myocardium of cardiac CT images, making it easier to assess myocardial thickness and shape consistently and more accurately in fatal coronary heart disease and hypertrophic myocardium. The latter case was also examined by Hu et al. (2023), who has devised a complete automatic segmentation method based on intensely supervised networks and 3D active shape models (3D-ASM) to outline left and right ventricles on cardiac magnetic resonance. Such methods favor better ventricular mass and ejection fraction measurements, which are important indicators in diagnosing systolic dysfunction.

Kwan et al. (2024) investigated DL-based analysis of myocardial strain. The analysis helped to identify early signs of minor contractile dysfunction, which is common in heart failure with preserved ejection fraction (HFpEF) or in patients treated with chemotherapy who develop a chemotherapy-induced cardiomyopathy. Sun et al. (2024) utilized a 4D flow MRI with DL-based segmentation and flow quantification to measure the volumetric consistency of the flow and improved evaluation of hemodynamic defects in congenital and valvular heart diseases. Advancements in motion tracking and registration have also augmented DL in cardiac imaging. DeepMesh is a mesh-based DL model that tracks the motion of the heart, providing a fine-grained representation of myocardial contraction (Meng et al., 2023). Upendra et al. (2023) utilized vision transformers to perform deformable image registration of cine cardiac MRI to monitor regional wall motion abnormalities (RWMA). Hossbach et al. (2023) used the k-space-based quantification of motion to correct MRI motion to enhance the fidelity of the images, especially in the cases of noncooperative/arrhythmic patients.

### 1.5. Rationale and objectives of this review

Despite a growing body of literature exploring ML for heart disease prediction, existing research is often fragmented across different datasets, techniques, and evaluation metrics. While several reviews exist, most are either outdated or limited in scope. For instance, Bhavekar et al. (2024) provided a semantic review of optimization and ML models for heart disease detection, focusing mainly on algorithmic comparisons rather than clinical integration or explainability.

Considering the rapidly evolving landscape, there is a pressing need for a comprehensive, up-to-date synthesis of the field that not only evaluates algorithmic performance but also considers issues of clinical applicability, model interpretability, dataset diversity, and ethical implications.

The primary objectives of this systematic review are as follows:

- To synthesize current research on ML approaches used in heart disease prediction across different cardiovascular conditions, including CAD, congenital heart disease, and heart failure.
- Comparing the performance of various ML algorithms based on evaluation metrics such as accuracy, sensitivity, specificity, F1 score, area under the receiver operating characteristic curve (AUC-ROC), and interpretability.
- To analyze the nature of datasets used in these studies, including their size, source, features, preprocessing methods, and availability (open source vs. proprietary).
- To highlight best practices in feature selection, model validation, and deployment strategies.
- To examine the role of XAI in enhancing trust and transparency in predictive models.
- To identify gaps and limitations in current research and provide future directions for improving the reliability, generalizability, and clinical relevance of the ML models in cardiology.

Moreover, this review draws from a diverse set of recent studies, including those focusing on traditional clinical features (e.g., age, cholesterol, and blood pressure), noninvasive biometrics (e.g., tongue features), and real-time signals (e.g., ECG). For instance, Sourov et al. (2025) proposed an XAI-based cardiovascular risk assessment system that incorporates transparency into deep models using SHAP and LIME visualizations, contributing to accuracy and interpretability.

Ultimately, this review seeks to bridge the gap between ML innovation and clinical cardiology by offering a roadmap for developing next-generation decision support systems that are accurate, interpretable, and integrable within clinical workflows. This review is a resource for technical researchers and healthcare professionals aiming to leverage AI to combat the global burden of heart disease.

## 2. Methods

### 2.1. Search strategy and information sources

A systematic and rigorous search strategy was adopted to ensure a comprehensive and unbiased review of the literature on the application of ML in heart disease prediction. The strategy followed the preferred reporting items for systematic reviews and metaanalyses (PRISMA) guidelines to maximize the inclusion of relevant studies and to maintain transparency and reproducibility throughout the process as shown in Algorithm.

The literature search was conducted across multiple academic databases known for their extensive coverage of peer-reviewed scientific and technical publications. These databases included PubMed, Scopus, IEEE Xplore, ScienceDirect, and SpringerLink. In addition, Google Scholar was utilized to capture gray literature and ensure no potentially significant articles were missed due to indexing issues. The time window for the search was set from January 2015 to July 2025, considering the rapid advancements in ML methodologies and their increasing integration into healthcare applications over the past decade.

The following search terms and Boolean operators were used to frame queries:

- “heart disease” OR “cardiovascular disease” OR “coronary heart disease”
- AND “machine learning” OR “DL” OR “artificial intelligence”
- AND “prediction” OR “diagnosis” OR “classification” OR “risk assessment”

These keywords were applied to the title, abstract, and keywords fields of the databses. To enhance search precision, filters were applied to include only English-language articles and to exclude nonpeer-reviewed sources such as blog posts and editorials. Additionally, snowball sampling was conducted on the reference lists of identified articles to locate further relevant studies not captured in the initial search.

### 2.2. Eligibility criteria (inclusion and exclusion)

A clear set of inclusion and exclusion criteria was established to guide the selection of studies for this review. These criteria ensured that only studies relevant to the research objectives were included.

#### Inclusion criteria

- Studies that explicitly applied ML algorithms (e.g., decision trees, SVMs, RFs, neural networks, etc.) to predict heart disease or its risk factors.
- Studies presenting empirical results, including accuracy, precision, recall, F1 score, and AUC-ROC metrics.
- Articles published in peer-reviewed journals or conference proceedings.
- Studies that used clinical, demographic, ECG, imaging, or laboratory data for modeling.
- Publications written in English between January 2015 and July 2025.
- Both traditional ML and hybrid models (e.g., ML with feature engineering, optimization, or XAI) were included.

#### Exclusion criteria

- Articles focused solely on data preprocessing or feature extraction without applying any ML model for prediction.
- Reviews, opinion pieces, editorials, and metaanalyses without new model development or testing.
- Studies that applied ML to other cardiovascular conditions did not directly involve predictive modeling for heart disease (e.g., arrhythmia detection without heart disease classification).
- Papers lacking sufficient methodological transparency or missing performance metrics.
- Duplicate articles from multiple databases.
- To ensure robustness, the inclusion and exclusion criteria were independently validated by 2 reviewers. Any discrepancies in study eligibility were resolved through consensus or consulting a third reviewer.

### 2.3. Study selection process

The study selection process adhered to the PRISMA guidelines and was conducted through a systematic, 4-stage procedure encompassing identification, screening, eligibility, and inclusion.

#### • Identification phase

In this initial stage, a comprehensive search was conducted across multiple academic databases and supplemented with manual searches to capture a wide range of potentially relevant studies. All records retrieved from the automated and manual searches were aggregated, and duplicate entries were removed using reference management software and manual cross-verification to ensure accuracy and prevent redundancy.

#### • Screening phase

Two qualified reviewers independently reviewed the titles and abstracts of the remaining unique records. The screening was performed to filter out studies that did not meet the predefined inclusion criteria. Disagreements between reviewers during this phase were resolved through discussion or consultation with a third reviewer to maintain consistency and objectivity.

#### • Eligibility phase

Full-text versions of the shortlisted studies from the screening phase were assessed in detail to evaluate their eligibility based on specific inclusion and exclusion parameters. These parameters included methodological rigor, use of ML models, proper dataset usage, and completeness of reported results. This phase ensured only methodologically sound and relevant studies advanced to the final stage.

#### • Inclusion phase

After full-text assessment, the final set of studies that met all inclusion criteria was compiled for detailed analysis in the review. Throughout this process, reasons for exclusion at each step were documented meticulously. To maintain the reliability of the selection process, interrater agreement was calculated and found to be substantial, further supporting the integrity of the review methodology.

### 2.4. Data extraction and quality assessment

The data extraction process of this systematic review was carefully constructed to achieve the comprehensive, consistent, and unbiased extraction of available methodological, clinical, and technical data of the studies passing the final criteria to be included. A data extraction form in Microsoft Excel was created that was standardized and structured so the important aspects of the study could be tabulated and analyzed. This form of extraction was piloted on a sample of randomly chosen articles (5 articles) before it was fully deployed. The preliminary test was useful in the design of the layout of the form, the elimination of redundancies, and the assurance that all relevant information could be adequately and proficiently recorded in the enormous scope of ML research into the prediction of heart disease. The final data extraction form consisted of 5 broad categories that considered the methodological strength, clinical significance, and predictive power of each study included in the data:

- Study identifier and metadata: The simplest of bibliographic data was recorded, including the name of the first author, title of the article, year of publication, journal, and the place or country of origin. This information was contextually detailed for geographical trends of research output and allowed us to assess the regional availability of data or statistical models.
- Dataset characteristics: It was vital to extract detailed information about the dataset used to determine reproducibility and generalizability. This included information on the name of the dataset (e.g., Cleveland Heart Disease dataset, Framingham Heart Study, UCI repository, or any other collection from Kaggle), the sample size, patient demographics, number and type of features (e.g., clinical, demographic, imaging, ECG signals), and distribution of classes (e.g., balanced or imbalanced). The focus was on whether the datasets used were publicly or privately available, synthetic or real life. This strongly influences the models developed, replicability, and external validity.
- ML models and techniques: The ML algorithms used in each study were described in detail. This included classical ML models (decision trees, SVM, RF, k-NN, and LR), ensemble methods (XGBoost, AdaBoost, and GBMs), and DL models (including DNNs, CNNs, and RNNs). Novel architectures or optimization methods (e.g., evolutionary algorithms, hyperparameter tuning, and metalearning) were also recorded. The completion of a model with hybridization was also listed, where applicable. The reason for model choice was also noted, as well as anything about model interpretability or deployment.
- Performance metrics and statistical justification: To facilitate a comparative evaluation of the model efficacy, performance outputs were produced with accuracy, precision, recall, sensitivity, specificity, F1 score, and AUC-ROC. More importantly, the review also reflected the existence and forms of validation methods adopted, including hold-out validation, k-fold validation, stratified samples, or the application of external validation solutions. Studies that had a weak validation mechanism were pointed out to be methodologically lacking. Moreover, we also retrieved the data on whether the statistical significance tests (e.g., t-tests, ANOVA, or confidence intervals) were supplied to support the recorded findings.
- Interpretability tools and explainability: Since, in the context of clinical ML, there is an increasing interest in XAI, we placed on record the application of interpretability algorithms like SHAP, LIME, Grad-CAM, and attention heatmaps. It was documented how far the authors were trying to make their model outputs clinically understandable or whether they were cooperating with experts in the field and verifying their predictions, which heavily affects the translational potential of the ML models in a medical setting.

Two well-versed reviewers in ML and healthcare analytics conducted data extraction independently. This dual review approach minimized individual bias and improved consistency. Any discrepancies in the interpretation or documentation of study features were resolved through detailed discussion and consensus. The corresponding authors were contacted via email to clarify persistent ambiguity or missing data. Where no response was received, the missing information was documented as “not reported”.

### 2.5. Quality assessment strategy

In order to assess the internal and external validity of the included studies, a modified version of the Newcastle–Ottawa scale (NOS) was used. This tool was initially designed to be used for observational studies. After incorporating components of the prediction model risk of bias assessment tool (PROBAST) framework designed explicitly for predictive modeling, NOS is now used in ML-driven medical research. The aim was to evaluate methodological rigor, reproducibility, clinical relevance, and bias control.

There were 5 primary aspects to consider when evaluating each study:

- Quality of the dataset: Studies were reviewed depending on the completeness, richness, and source of the dataset utilized. Publicly released datasets with well-documented characteristics were valued as high as proprietary or synthetic datasets lacking sufficient disclosures. The sample sizes, class balances, and feature diversity were considered.
- Model validation strength: Most of the studies focused on this aspect. Cross-validation, separate test datasets, and overfitting avoidance were perceived to be signs of good modeling. Studies that only provided results of training accuracy or did not test their models on unknown data were ranked low.
- Reproducibility and transparency: Transparency played an important role in this review. Studies that uploaded their codebase (e.g., on GitHub), offered pseudocode of the algorithms, or a full breakdown of the model configuration and preprocessing protocol were marked as more transparent and scientifically plausible. Inaccessibility to implementation information became a severe obstacle to replication.
- Bias mitigation and overfitting control: This criterion looked at investigations of how a study handled class imbalance, data leakage, or overfitting. Methods such as synthetic minority over-sampling technique (SMOTE), under sampling, and increasing data via augmentation, feature selection, and dropout layers in the neural net were noted. Studies that failed to mention how they dealt with these challenges were deemed to have a higher probability of bias.
- Clinical relevance and domain integration: Finally, the degree of collaboration with healthcare professionals, use of real-world clinical data, and contextualization of results for medical practitioners were assessed. Studies that evaluated their models in a clinical setting or incorporated expert feedback were favored for their practical applicability.

By integrating quantitative and qualitative assessment tools, this review ensured that only high-quality, rigorously validated, and clinically relevant studies were included in the final synthesis. The methodological thoroughness supports the credibility of the conclusions drawn. It helps identify best practices for developing and deploying ML-based heart disease prediction tools in real-world settings.

## 3. Results

### 3.1. Overview of included studies

The study selection process adhered to the PRISMA guidelines to ensure a transparent and reproducible synthesis of the literature. The multistage process, detailed in the PRISMA flow diagram (Figure), systematically filtered the literature to identify studies pertinent to the application of ML in cardiovascular disease prediction. The initial database search yielded 2507 records. After the removal of duplicates, 1787 unique articles remained for the screening phase.

In the screening phase, 2 reviewers independently assessed the titles and abstracts of the 1787 records against predefined inclusion criteria. This stage aimed to filter out studies that were clearly irrelevant. A total of 1289 records were excluded, primarily because they did not focus on the application of ML for heart disease prediction, lacked original empirical data (e.g., review articles, editorials, or opinion pieces), or did not constitute predictive modeling research.

The remaining 498 articles proceeded to a full-text eligibility assessment. Each paper was thoroughly reviewed to evaluate its methodological rigor, dataset characteristics, validation procedures, and the completeness of its reported results. This detailed evaluation led to the exclusion of an additional 433 articles. The predominant reasons for exclusion at this stage were significant methodological limitations, such as the absence of robust validation techniques (e.g., k-fold cross-validation); failure to report essential performance metrics required for comparative analysis; insufficient transparency regarding the ML pipeline, which would impede reproducibility; or the use of private, undocumented datasets.

Upon completion of the eligibility assessment, a final cohort of 65 studies met all inclusion criteria. These studies were deemed to be of sufficient methodological quality and relevance to be included in the final qualitative synthesis, forming the evidence base for this systematic review.

### 3.2. Datasets for heart disease prediction

#### 3.2.1. Publicly available datasets

Publicly available datasets have formed the cornerstone of research in heart disease prediction using ML. These datasets offer structured, labeled data that enables benchmarking and cross-comparison of algorithms.

Table 1 presents a comparative overview of widely used online heart disease datasets, detailing their core characteristics such as the number of features, total instances, and the distribution of healthy and unhealthy cases. Datasets like Cleveland, Hungarian, and Statlog have been pivotal in developing classification models, whereas larger datasets like Framingham offer more extensive instances for statistical learning. Reviewer ratings and justification columns offer an expert evaluation of dataset quality, diversity, and usability. This overview is a foundational reference for selecting appropriate datasets based on research needs. A more detailed analysis of each publicly available dataset is presented in the subsequent sections.

Table 2 illustrates a detailed comparison across other state-of-the-art datasets for cardiovascular detection and their respective analysis of strengths and limitations.

##### A. UCI Cleveland heart disease dataset

Among the most frequently used datasets is the UCI Cleveland heart disease dataset, which includes 76 attributes but typically uses a subset of 13–14 features relevant to heart disease diagnosis. This dataset has been central to many foundational studies (El-Sofany, 2024; Bhavekar et al., 2024). The Statlog (Heart) dataset, derived from the Cleveland dataset, contains 270 observations and is often used in traditional ML pipelines such as LR, SVMs, and decision trees (Rani et al., 2024b). Originating from the Cleveland Clinic Foundation, this dataset is available through the UCI ML repository and has served as a foundational dataset in classification and feature selection tasks.

Dataset characteristics:

- Source: UCI ML Repository.
- Total Samples: 303 patient records.
- Number of Features: 13 input features + 1 target variable (showcased in Table 3).
- Target variable: Diagnosis of heart disease (values 0–4), where 0 indicates no disease and 1–4 represents varying disease severity levels. For binary classification tasks, values 1–4 are typically converted to 1 (presence of heart disease).
- Missing values: Minimal missing data. Some entries (particularly for the ca and thal features) contain “?” placeholders, which require imputation or exclusion.

Numerous studies have used this dataset due to its manageable size and well-labeled features. Algorithms such as SVMs, decision trees, LR, and RFs have been tested extensively on this dataset. Feature selection techniques such as particle swarm optimization (PSO), recursive feature elimination (RFE), and PCA have also been applied to identify optimal subsets of predictors. The relatively low dimensionality makes this dataset suitable for quick prototyping and educational purposes. Despite its age, it remains a strong benchmark for classification performance comparisons across various models. However, the dataset presents significant limitations in terms of clinical generalizability:

- The small sample size (n = 303) may not capture broader population variability.
- Some features contain missing or ambiguous values (e.g., “?” in thal and ca).
- The dataset is decades old, with features reflective of historical diagnostic practices.
- Class imbalance: original target distribution is skewed toward the nondisease class (0).

##### B. Kaggle heart disease dataset

The Kaggle heart disease dataset is a recent compilation of features from multiple publicly available heart disease datasets, including the Cleveland, Hungarian, Swiss, and Long Beach VA datasets. This dataset is curated to present a clean, preprocessed version, often used in data science competitions and educational platforms.

Dataset characteristics:

- Source: Kaggle (user-contributed dataset, based on public data).
- Total samples: Approximately 1025 (aggregated and cleaned).
- Number of features: 11 input features + 1 target variable (shown in Table 4).
- Target variable: HeartDisease (binary: 1 = disease, 0 = no disease).
- Missing values: None (data is precleaned).

The strengths of this dataset lie in its precleaned nature and balanced class distribution. It enables researchers to focus more on model development than on intensive data preprocessing. A broad range of models, such as XGBoost, LightGBM, ANNs, and DL architectures, have been tested using this dataset.

Depending on model requirements, the categorical features are often label-encoded or one-hot encoded. The relatively large sample size improves generalizability compared to the UCI Cleveland dataset.

However, the dataset presents significant limitations:

- Although larger than the UCI dataset, the dataset is derived from older public repositories.
- There is limited transparency regarding the merging methodology and preprocessing pipeline.
- The absence of longitudinal or temporal features restricts its use for progression modeling.

Despite these limitations, the Kaggle heart disease dataset is a valuable midsize dataset for experimental evaluations.

##### C. Framingham heart study

The Framingham heart study (FHS) is a renowned longitudinal epidemiological study that began in 1948 in Framingham, Massachusetts, USA. The Framingham dataset used in ML is typically a sanitized, deidentified version of offspring or third-generation cohorts, focusing on 10-year risk prediction of coronary heart disease (CHD). It includes longitudinal data from a community-based cohort. This dataset enables risk prediction modeling over time, capturing age, cholesterol, blood pressure, and smoking status variables. It supports classification and regression-based models due to its temporal nature (Pandey et al., 2024).

Dataset characteristics:

- Source: National Heart, Lung, and Blood Institute.
- Total samples: 4240 patient records.
- Number of features: 15 (shown in Table 5).
- Target variable: TenYearCHD (1 = risk present, 0 = no risk)
- Missing values: Minor (less than 5%), typically imputed using mean or k-NN imputation.

This dataset is particularly valued for its longitudinal nature and demographic diversity. Its relatively large sample size and feature richness make it suitable for traditional classifiers and complex models like DL or time-series prediction. Studies have utilized this dataset to develop risk stratification models, often comparing their performance against traditional scoring systems such as the FRS (Rimal et al., 2024; Zhang and Mu, 2024). This dataset includes lifestyle variables such as smoking and BMI, supporting public health modeling and clinical diagnostics. The dataset also has some evident limitations:

- The target variable indicates risk (not actual disease), which can misrepresent outcome accuracy.
- The dataset lacks imaging data and more granular ECG or EHR information.
- Certain categorical features like “education” are prone to being underrepresented or inconsistently encoded.
- Due to its American origin, its applicability to non-US populations requires careful cultural and healthcare context alignment.

##### D. Statlog heart disease dataset

The Statlog heart disease dataset has numerous applications as a benchmark dataset for binary classification allocation tasks regarding heart risk forecast. It contains 270 cases and 14 characteristics, both collected qualitatively and numerically. The data used in this dataset is part of the original UCI heart disease dataset in a format that is used in ML with a special focus on LR, decision trees, and SVM learning. The dataset is relatively balanced, with 150 healthy and 120 unhealthy cases. It is well packaged with a small footprint and suitable for use as a model prototype and comparative algorithm research. Researchers often use the Statlog dataset as it is simple, readily available, and used to verify the classification algorithms of CVDs.

The information in Table 6 contains the main characteristics of the Statlog heart disease dataset, used in many classification problems of CVDs. It has 14 variables, including continuous and categorical variables. Clinical measurements include resting blood pressure, cholesterol, and the maximum heart rate reached, as well as demographic information covering age and sex. The binary indicators, including fasting blood sugar, exercise-induced angina, and heart disease, are also detected in the dataset.

The presence or absence of such features as the type of chest pain, the result of ECG at rest, the slope of the ST segment, and thalassemia offers more informative details concerning the health status of the heart. Clinical granularity is provided by additional physiological measures, such as the number of vessels with fluoroscopically determined coloration and the ST depression caused by exercise. The characteristics collectively facilitate a powerful training and testing of ML models that should occur in the context of heart disease diagnosis and prediction.

#### 3.2.2. Clinical and EHR datasets

A more practice-oriented view can be derived from clinical data collected in hospitals, research centers, or national health databases. Such data include high resolution ECG and echocardiograms, laboratory results, demographic patterns, and medical annotations. Several studies have also used hospital-specific data, including the dataset of the Jordan University Hospital (Alshraideh et al., 2024), which included various clinical features used to train ensemble classifiers. EHRs can contain longitudinal and multimodal data, making them suitable for temporal and fixed conditions (Ma et al., 2024). These datasets can be used for progression modeling and outcome prediction for myocardial infarction, mortality, or readmission. Wearable data and in-the-moment observations, including mobile cloud scenes, are becoming increasingly popular (Saheed et al., 2024). Nonetheless, the privacy issues, nonuniformity of data formats, and the lack of values pose considerable preprocessing and methodological challenges (Mesquita and Marques, 2024).

#### 3.2.3. Key features and data types

The quality and diversity of the input features are the main factors defining the prediction performance of an ML model. Standard variables comprise demographic characteristics (e.g., age and sex), lifestyle factors (e.g., smoking and physical exercise), and clinical variables (e.g., blood pressure, cholesterol level, and heart rate). Laboratory data such as lipid profiles, troponin, and glucose levels are also fundamental to feature sets (Islam et al., 2024). The features extracted by signals, especially ECGs, have played a central role in boosting the prediction accuracy.

Time- and frequency-domain features that are identified through the application of signal processing algorithms are frequently fed into DL concentrated models such as CNNs (Pachiyannan et al., 2024). Other new data types involve tongue image characteristics (Duan et al., 2024) and gene expression. The next vital step is feature engineering. This includes normalization, discretization, and dimensionality reduction methods (PCA or t-SNE). Genetic algorithms or mutual information strategies are used to fine-tune the input sets using automated feature selection (Mandula and Vijaya Kumar, 2024).

#### 3.2.4. Data balancing techniques and their significance

Class imbalance is a pervasive problem in CVD prediction, often inflating accuracy while masking poor sensitivity to minority (high-risk) cases. Oversampling methods such as SMOTE (Akinola et al., 2024) and its variants (Alamsyah et al., 2021) are shown in Table 7 . Synthetically enlarging the minority class can improve model discrimination. Undersampling approaches (e.g., near miss) reduce majority-class dominance but risk discarding informative samples. Hybrid schemes combine both strategies (Kumar, 2025). More recent generative-model techniques include GAN-based synthesis (Campello et al., 2022). Furthermore, variational autoencoders produce realistic minority instances, preserving data distribution.

Cost-sensitive learning embeds class-specific penalties directly into the loss function, obviating explicit resampling. Empirical studies across diverse CVD datasets (e.g., MIMIC-III, Framingham, UK Biobank) consistently show that appropriate balancing raises recall and F1 score without sacrificing specificity (Zhu et al., 2023). Ignoring these techniques leaves models vulnerable to biased predictions, undermining clinical trust and limiting deployment in heterogeneous patient populations.

### 3.3. ML algorithms for CVD classification

ML has emerged as a pivotal tool in CVD classification, enabling early detection, risk stratification, and long-term prognostic assessment. This section explores the application of ML algorithms in CVD prediction, segmented into 3 subcategories based on their functional characteristics: classification models, regression-based models, and hybrid or comparative approaches.

- Classification models primarily categorize patients into risk groups (e.g., disease vs no disease) using labeled datasets. These include algorithms such as SVM, decision trees, and ensemble methods like RF and GBM.
- Regression-based models predict continuous outcomes, such as cholesterol level or blood pressure over time, and can be vital for individualized treatment planning. Linear and polynomial regression are traditional techniques, while ML enhances these with adaptive and nonlinear capabilities.
- Hybrid and comparative studies combine multiple models to improve accuracy, robustness, and interpretability. These studies often benchmark ML algorithms like XGBoost, LightGBM, and DL networks against traditional statistical methods or one another to validate performance across diverse datasets.

This structured categorization comprehensively explains how different ML models improve CVD diagnosis, prognosis, and treatment planning.

As illustrated in Table 8, ensemble learning methods like RF, GBM, and XGBoost, have considerably improved the accuracy of predictions by integrating several weak learners. García-Ordás et al. (2023) used ensemble learning and feature augmentation to predict heart disease using robust classification measurements. The functioning of the models is particularly efficient with high-dimensional biomedical data, which Khanna et al. (2023) used when combining ensemble classifiers and ECG signals produced by internet of things (IoT) disease detection. In the same study, RF and XGBoost were used in smart home health monitoring systems to predict type 2 diabetes and hypertension. The conclusions of their observations indicate that ensemble-based methods increase classification rates and potentially increase the applicability of the model in more varied demographic settings.

#### 3.3.1. Classification-based studies

The most popular goal in modeling heart diseases via ML is classification. The aim is to sort people into groups, such as those with CAD or not. Accuracy, precision, recall, F1 score, and AUC-ROC are metrics used in assessing performance (Rehman et al., 2025). In this review, we focused on studies that classified heart diseases using ML techniques (Table 9).

Studies have reported optimized ensemble methods with classification accuracies over 90% (Islam et al., 2024; Ogunpola et al., 2024). For example, Bouqentar et al. (2024) obtained 97.2% accuracy using a combination of feature engineering and an RF model. Another comparable study showed the successful application of DL (CNNs) in feature-intensive conditions, with an AUC of 0.96 based on ECG-derived data (Rani et al., 2024b). Nevertheless, model bias, interpretation, and imbalance of data pose significant challenges. Data balancing using techniques such as SMOTE, and explainability (e.g., SHAP values) also get incorporated into pipelines to a greater degree (Pandey et al., 2024).

Classification methods have been applied quite extensively in heart disease predictions to determine whether there is a disease or not. The effectiveness of supervised ML models has been proven many times over, with LR, SVM, RF, and XGBoost being some of the most used. For example, Bhatt et al. (2023) conducted a comparative analysis of several algorithms and found that RF and XGBoost-based methods showed better classification accuracies and robustness. In a similar manner, Jain and Agrawal (2023) showed that SVM, LR, and XGBoost classifiers delivered comparable performance in predicting heart failure. These studies emphasize the utility of ML-based classification in diagnostic practice (Ansari et al., 2023). They also confirm that ML-based classifiers trained on carefully sampled datasets can achieve high precision and recall rates, and F1 scores can reach 90% or more.

ANN, CNN, and RNN are DL neural networks that can model nonlinear relationships. They have become the gold standard in health risk classification. A review by Kaul et al. (2022) addressed how DL can transform the healthcare analytics environment by improving classification accuracy of different clinical patterns. García-Ordás et al. (2023) applied enriched DL models using feature augmentation strategies that drastically changed heart disease detection rates. In a related case, Srikanth et al. (2023) suggested an adversarial DL system uniquely adapted to identify particular substructures in brainstem MRI scans, highlighting the power of DL in high-resolution imaging. The use of deep networks in wearable health technologies has also been considered (Khanna et al., 2023). Masoumian Hosseini et al. (2023) considered smartwatches and biomedical signal processing, where deep model integration can diagnose disease in real time. RNNs have been very effective with temporal analysis, and CNNs with spatial feature extraction, especially in predicting chronic diseases. Although these architectures are data intensive, they are excellent for feature representation and automatic learning compared to traditional and ensemble approaches.

The practical application of ANNs has become very popular, especially the multilayer perceptron, due to their capacity to consider nonlinear dependencies. More evolved structures, such as CNNs, are used in signal and image-based data, ECG, and echocardiography (Majhi and Kashyap, 2024). RNN and longer short-term memory (LSTM) networks have performed well in sequential data modeling of EHRs for temporal prediction (Saheed et al., 2024). A hybrid model that combines DL and classic ML classifiers is becoming a new paradigm. Babu et al. (2024) discussed the concept of quantum-enhanced neural networks, where high complexity classification tasks can be achieved with performance improvements. With DL, explainability is a point of concern. Attention mechanisms, SHAP, and LIME are becoming more common in an attempt to explain model decisions and make them more acceptable in a clinical context (Mesquita and Marques, 2024). Attention-based frameworks have been used to classify *Mycoplasma pneumoniae* (Banerjee et al., 2025a). The Harris Hawks optimization based novel UNet-inception attention architecture (HHO-UNet-IAA) is an explainable attention feed-forward setup that has been used for glaucoma segmentation (Banerjee et al., 2025b; Singh et al., 2025b). Attention mechanisms in organ segmentation of thoracic imaging was refined through resio-inception U-Net with deep cluster recognition (Saminathan et al., 2024), highlighting the potential of dynamic testing and attention-based XAI solutions. For breast cancer classification, the CICADA (UCX) model delineated aggressiveness effectively using hybrid architectures (Singh et al., 2025c, 2025a) using attention-based analysis of predictive modeling approaches. The analysis of methodological advances in ML for cardiovascular disease classification and segmentation is shown in Table 10 .

Model performance has also improved because of feature selection and optimization techniques. Saranya and Pravin (2023) combined the sensitivity of features and correlation, which greatly increased the accuracy of predictions. Chandrasekhar and Peddakrishna (2023) addressed hypertuning metaheuristics to optimize the performance of ML classifiers in another direction based on optimization. Other experiments have shown that hybrid classifiers using SVM and ensemble learners can improve detection, particularly when given imbalanced data (Hani et al., 2022; Khan et al., 2023). In general, classification models for predicting heart disease are well developed, with accuracy more than 90% on average across all the models, AUC between 0.90 and 0.97 on average across all the models, and stable F1 scores.

Table 10 presents a consolidated overview of selected studies focused on the classification of CVD using ML and DL techniques. The listed studies explore various classification tasks, including binary classification (presence or absence of heart disease), multiclass diagnosis, and severity grading. Each method has distinct strengths such as addressing data imbalance, enhancing interpretability, or improving prediction accuracy. Table 10 also highlights the specific limitations that each study overcame, along with reviewer-assigned ratings based on methodological rigor, clinical relevance, and innovation. These studies illustrate the progressive advancement of classification models in the healthcare domain and their growing applicability in early CVD detection and decision support.

##### 3.3.1.1. Performance metrics for CVD

Beyond overall accuracy, CVD models must report metrics that reflect clinical relevance. Recall (sensitivity) quantifies the proportion of correctly identified disease cases—a critical safety measure for screening tools. Precision (positive predictive value) balances false-positive burden, essential for resource-constrained settings.The F1 score harmonizes recall and precision, offering a single indicator of trade-off performance. AUC-ROC evaluates discrimination across thresholds, while area under the curve precision recall (AUC-PR) is more informative under severe imbalance.Calibration metrics (e.g., Brier score and calibration plots) assess agreement between predicted probabilities and observed outcomes, a prerequisite for risk stratification.Decision-curve analysis translates metric performance into net clinical benefit, guiding threshold selection.Recent CVD studies emphasize reporting net reclassification improvement and integrated discrimination improvement when adding novel biomarkers or imaging features. Comprehensive metric suites ensure that models are statistically sound and actionable in real-world cardiology practice.

##### 3.3.1.2. State of the art comparative study for CVD classification

The state of the art comparative study is detailed in this section and tabulated in Table 11.

Nancy et al. (2022) suggested an IoT-cloud-integrated smart healthcare monitoring device that uses DL models to forecast heart diseases. Their structure permits the uninterrupted collection of physiological indications of patients using IoTs, which are analyzed on a cloud platform using sophisticated DNNs. The study accurately indicates the scalability of cardiovascular condition diagnoses using cloud-enabled architectures to identify real-time diagnoses.

Barhoom et al. (2022) designed a comparative framework, combining several ML and DL algorithms to predict heart diseases. Their experimental findings showed that DL methods are better at recall (94%) than traditional ML classifiers. The research combines several models, highlighting the significance of the ensemble decision-making process in enhancing the reliability of the diagnosis.

Raju et al. (2022) developed a cascaded DL model embedded in an IoT and fog-computing infrastructure. Their model performed very well (94% accuracy and 98% precision) because they used a 2-tiered structure in which primary features were obtained in the IoT devices and refined on the fog nodes. The method is less latent and more predictive in clinical environments in real time.

A hybrid of an RNN and an LSTM model was tested by Bhavekar and Goswami (2022). Using the sequentiality of cardiovascular data, the hybrid architecture captured temporal dependencies in patient health records, making it applicable to the longitudinal prediction of heart diseases. Correspondingly, Goswami et al. (2022) used VGGNet on ECG signal classification to identify the strength of deep CNNs in medical signal interpretation.

Trying to predict heart disease, Mehmood et al. (2021) developed a deep convolutional neural network (DCNN) with a reported accuracy of 97%. This paper shows that CNN models successfully attain spatial patterns in medical signals, but precision and recall (86% and 81%, respectively) indicate the need to optimize them further. Xiao et al. (2020) used DL to segment the coronary artery and provide risk warnings with good precision (91%) but a low recall (66%). This means the model was more efficient in eliminating false positives than in identifying actual cases.

Ali et al. (2020) made a step forward in developing an ensemble DL model with feature fusion to monitor competent healthcare. Their framework combines heterogeneous features, enhancing the generalizability to various datasets. Similarly, Mienye and Sun (2021) reported a better ensemble learning model to predict the risk of heart disease with 93% accuracy, supporting the idea that ensemble models are much better than single classifiers.

Pan et al. (2020) executed a high-ranking DL-based CNN in the internet of medical things (IoMT). Their method was claimed to be almost perfect (99%), which shows the promising prospects of its use in a real-life scenario in a wearable and sensor-based healthcare ecosystem. Further, attention is given to the application of IoT, suggesting the use of a multidimensional CNN (MDCNN) classifier in the framework of IoT that gave a 98% accuracy, which supports the usefulness of combining IoT and DL.

Ali et al. (2020) introduced an automated diagnostic system based on an optimally configured DNN with statistical modeling, and achieved an accuracy of up to 100% and a recall rate of 85%. HealthFog is a fog-computing AI heart disease diagnostic healthcare system (Tuli et al., 2020). The system proved extremely accurate (98%) and showed the benefits of placing calculation nearer to the data sources to increase efficiency.

Hassan et al., (2023) used transfer learning by using pretrained DNNs and dimensionality reduction via PCA. Their experiment showed that model fine-tuning based on PCA would not only increase the computational efficiency but also increase the diagnostic accuracy especially in high-dimensional data. Patro et al. (2021) suggested a new optimization algorithm to use in supervised learning to predict heart diseases; however, deeper performance measures were not consistent across benchmarks.

Sharma and Parmar (2020) showed that simple DNN architectures can be effective. They used DNN to predict heart diseases with 90% accuracy. Grey wolf optimization (Al-Tashi et al., 2018) and PSO-based stacked autoencoders (Mienye and Sun, 2021) are optimization-based methods to improve feature selection and classification. Al-Tashi et al. (2018) had a specificity of 91% and a recall of 93%, whereas Mienye and Sun (2021) had better overall performance (97% accuracy, 94% precision, 100% recall, and 97% F1 score), which shows the robustness of optimization in classification.

#### 3.3.2. Regression-based studies

Regression models are less common, but still important for predicting a continuous outcome variable such as a risk score, survival probability, or physiological measurement. The performance of such models is frequently determined on the basis of measures such as root mean square error (RMSE), mean absolute error (MAE), and R^2^ (Ma et al., 2024). Framingham-based research typically uses Cox proportional hazards models with ML improvements to estimate long-term cardiovascular danger (Chinni and Manlhiot, 2024). Systolic blood pressure and cholesterol can also be estimated using linear regression and regularization methods with demographic and lifestyle features (Zhang and Mu, 2024). Regression models are especially useful for clinical decision support systems where it is more informative to quantify risk rather than to classify it. However, they have not been as widely utilized compared to classification methods. Logistic regression, though simplistic, is widely used because of its explainability and suitability for binary classification tasks (Bouqentar et al., 2024). Decision trees offer hierarchical rule-based classification and have the advantage of visual interpretability. SVMs have also been extensively applied due to their robustness in handling high-dimensional data and their effectiveness with kernel functions (Türk, 2024).

Naive Bayes has shown utility in lightweight predictive modeling, particularly in resource-constrained settings or as a component in ensemble strategies (Zhang and Mu, 2024), despite the assumption of independence. These models often form the baseline for comparative analysis, helping to evaluate the performance improvements gained from more complex architectures.

The standard ML algorithms used in disease risk prediction and classification are LR, SVM, decision trees, and naive Bayes. Thatha et al. (2025) used LR and SVM to study the risk of cardiovascular and metabolic diseases with a focus on interpretability of these models in clinical decision making. Similarly, a recent experiment tested the performance of SVM and decision tree classifiers in long-term prediction of CAD, with good accuracy and transparency of the decision boundaries (Trigka and Dritsas, 2023). These models are especially popular when interpretation and computational efficiency are more important than raw performance. Dritsas and Trigka (2023) verified traditional supervised models on liver disease data, showing that a small number of highly valuable features can yield competitive performance, even in a low-resource setting. In another attempt to validate naive Bayes and LRs, Guarneros-Nolasco et al. (2021) determined certain risk factors of CVDs, emphasizing the importance of simplicity in a risk pipeline.

Despite not being discussed as frequently as classification, regression models significantly operationalize continuous clinical outcomes. They predict health-related risk scores, time to onset, and disease improvement. Patient-specific cardiovascular risk scores can be estimated in the framework proposed by Kumar et al. (2024) that used the random forest and logistic regression grid (RF-LRG) algorithm and cloud assistance. Their system was exact in regression and flexible toward real-time edge conditions. Regression modeling has been enhanced using advanced optimization techniques. The slime mold algorithm (Li et al., 2020), pathfinder algorithm (Yapici and Cetinkaya, 2019), and forest algorithm (Wu, 2014) have been used to reduce hyperparameter tuning and improve generalization during continuous output prediction. Although AI has been used to calculate the probability of heart failure (Choi et al., 2020), very few tools have implemented biomarkers and imaging features into regression models. However, they are of great clinical utility, particularly regarding risk stratification and prognosis modeling. In this review, we focused on the regression models of heart diseases that use ML techniques, where regression was used to predict continuous risk scores or clinical indicators (Table 12).

#### 3.3.3. Hybrid and comparative studies

An increasing body of research follows the hybrid modeling paradigm, which applies classification and regression to take advantage of both paradigms. Karna et al. (2025b), featured in Table 13, used a 2-tier approach in which patients were initially sorted into 2 risk categories using a classification model, and the risk scores were estimated using a regression model. Comparative studies that benchmark the performance of various algorithms on a common dataset are increasing too.

Ensemble learning has been particularly effective in the prediction of heart diseases. RF combines the results of many trees to get better results and minimize overfitting. GBMs, especially of XGBoost, perform well because of their ability to handle missing data, regularization, and scalability (Omkari and Shaik, 2024). Experiments on ensemble models have consistently outperformed single estimators, given that they are combined with feature selection and hyperparameter optimization procedures (Rimal and Sharma, 2024). For example, HXAI-ML, is a hybrid explainable model that integrates RF with DL methods and has demonstrated high accuracy and interpretability (Talukder et al., 2025).

These usually entail an AutoML pipeline or metaheuristic optimization (Sourov et al., 2025). These methodologies estimate the best model to use in a particular data scenario, and for what purposes (Rimal and Sharma, 2024). The trade-offs between performance and explainability favor hybrid methods. For example, methods blending inexpensive explainable models, such as LR, and more capable but still obscure models, such as DL, have found such a balance between trustworthiness and excellence (El-Sofany, 2024). The general trend in the literature is methodological pluralism. A dominant approach is lacking, but several strategies are adjusted to specific datasets, prediction targets, and deployment contexts.

Table 13 summarizes the use of hybrid and comparative studies to combine the strengths of classification and regression paradigms to achieve better predictive or clinical interpretation. Das et al. (2023) compared the performance of ML models to predict heart stroke has shown that classifier models (e.g., decision trees and RF) in combination with regression analysis performed better in decision-making within real-time systems. Similarly, Commonly used deep neural solutions for CVD segmentation

This section addresses the state-of-the-art DL methods that have significantly advanced the segmentation of CVD related structures in medical imaging. Accurate segmentation of cardiac and vascular regions from modalities such as MRI, CT, and ultrasound is vital for quantitative assessment and diagnosis of various cardiovascular conditions. DNNs, particularly convolutional architectures like U-Net and FCNs, have become the predominant solutions due to their ability to delineate complex anatomical features with high precision automatically. These methods improve segmentation accuracy and enhance clinical metrics such as ejection fraction and myocardial mass estimation. Innovations such as attention mechanisms, multiscale feature integration, and shape priors further refine segmentation outcomes. The deployment of such automated segmentation tools promises to reduce manual effort, minimize errors, and provide robust, reproducible analysis crucial for effective CVD diagnosis and management.

The myocardial MRI analysis pipeline developed for a DL framework is composed of several important phases that help to facilitate the correct interpretation of cardiac images. First, this pipeline begins with acquiring images, whereby raw MRI images of the heart are obtained in standard, common formats (DICOM or NIfTI), allowing compatibility with the rest of the computational processes. The preprocessing stage includes a few obligatory steps: loading image data with the help of special libraries (e.g., pydicom or nibabel), subsequent resampling to obtain a consistent spatial resolution, and the normalization of pixel intensities to provide the consistency of the image contrast. In order to improve on the better aspects of the image, noise reduction algorithms like the Gaussian filter or nonlocal means denoising methods are used. This removes the artifacts to a large extent, while retaining the important details about anatomical structure. They proceed with segmentation intended to define applicable cardiac structures, generally using CNN or similar supervised learning models trained on Keras. In each segmented region, feature extraction is performed, where the morphological and functional parameters of each region are measured to be analyzed further. Data augmentation and cross-validation measures are incorporated throughout the pipeline to increase model generalizability and ensure they are not overfit.

CardSegNet, a hybrid network of CNNs and vision transformers, can adaptively focus on both local and global information to increase the accuracy of segmentation in cardiac MRI (Aghapanah et al., 2024). Similarly, Portal et al. (2024) reported attention-based neural networks that segment the cardiac region, improving strain and volume prediction.

Numerous studies have discussed center and modality heterogeneity—two issues related to MRI (Arega et al., 2021). Brahim et al. (2022) used targeted data augmentation to enhance segmentation of the right ventricle in multicentered datasets. They developed an approach called ICPIU-Net that used the classification of priors to segment myocardial diseases precisely based on delay-enhanced MRI. Li et al. (2021) proposed MDFA-Net for multisequence magnetic resonance segmentation that integrates multiscale aggregation features with dual-path fusion of different features.

Guidance-based architectures have grown popular in polishing cardiac segregation. Using an attention-guided U-Net, Cui et al., 2021 developed a model that learns adaptive spatial focal attention to delineate centrally in short-axis MRI. Wang et al. (2023) used autoweighted supervision to label the combined myocardial scar and edema using multisequence data. Portal et al. (2024) also highlighted the advantages of attention in enhancing volumetric computations for cardiovascular segmentation.

Domain robustness is essential. Scannell et al. (2020) addressed this issue through domain-adversarial learning to preserve accuracy in segmenting different centers and vendor conditions. Kalapos et al. (2023) used deep networks to automate T1 and T2 mapping segmentation with high sensitivity in different patient conditions.

The change from transformer-based to federated learning is also apparent. Mazher et al. (2024) suggested a self-supervised bolster coordinate endpoint as a spatial and temporal transformer fusion phase aimed at 4-dimensional segmentation in federated learning conditions and empowered discretion-protective division training. Similarly, Khan et al., (2024) proposed a crop and couple approach through an interconnected network of specialists to improve segmentation quality in local and contextual perspective levels. Cross-modal and multimodal learning are major trends in predictive modeling. Karthikeyan (2024) introduced a cross-modal transfer learning architecture that used modalities to predict CVDs. Zhu et al. (2023) used a multibranch residual network, which could guess CVD by successfully fusing multimodal data. By integrating CNNs with classic ML, RF-CNN-F performed well in the prediction of CAD based on MRI (Khozeimeh et al., 2022). The value of CNN-RNN hybrids in prognosis and survival analysis was tested by Lu et al. (2023), who showed that sequential and spatial data could be integrated to be prognostic.

With more robust imaging configurations, Zhang et al. (2024) incorporated Fourier-enhanced features into a gradient-driven network, which showed the possibility of signal-level performance elevation of improved segmentation but is also applicable to the cardiac MRI pipeline. Other pioneering work on magnetic resonance segmentation using hybrid 2D and 3D fully convolutional networks was also documented by Patravali et al. (2018). Finally, more recent signal-based modalities, such as the Shannon energy-based algorithm of heart sound segmentation (Arjoune et al., 2024) highlight the increased importance of nonimaging physiological data in adequate heart monitoring and segmentation.

Table 14 presents a structured summary of recent influential studies exploring advanced ML and DL methodologies for medical image analysis and disease prediction. Each entry includes the authorship and publication year, title, primary research objective, methodology used, dataset used (with sample size, if specified), and a qualitative rating of the segmentation approach used.

#### 3.3.4. Use of AutoML for CVD

AutoML frameworks have emerged as effective tools for CVD prediction and classification that redefine the traditional analytics workflow by automating data preprocessing, feature selection, model selection, and hyperparameter optimization (Jiang et al., 2021; Rajeev and Natarajan, 2024; Budihal et al., 2025). Systems designed to automate ML, such as AutoGluon, H2O, and tree-based pipeline optimization tool (TPOT), reduce the requirement for advanced coding and statistical skills. Accessibility not only makes the research development process faster but also allows clinicians and domain experts to create robust predictive models to use in health informatics directly (Jiang et al., 2021; Rajeev and Natarajan, 2024; Wang et al., 2024).

Recent results highlight the better performance and generalizability of models with AutoML over past ML methods on a variety of heart disease data (Jiang et al., 2021; Rajeev and Natarajan, 2024). For example, AutoML ensemble pipelines can evaluate and optimize a set of algorithms (such as CatBoost, LightGBM, and XGBoost) and determine the best configuration without requiring much human intervention. The best-in-class results are: accuracy of over 91% and AUC values of up to 0.956 in CAD, and CatBoost-based AutoML can detect heart failure at 99.39% accuracy (Değer, 2024).

Importantly, AutoML systems are being developed to have closely coupled XAI modules. SHAP and LIME are used in these pipelines to provide model transparency, which is crucial for clinical credibility. The effects of clinical characteristics such as ST segment slope, character of chest pain, lipid indicators, and fasting glucose on automated decisions are explained, enhancing clinical trust and actionable interpretability (Değer, 2024; Rajeev and Natarajan, 2024; Wang et al., 2024; Sreeja et al., 2024; Islam et al., 2024; Majhi and Kashyap, 2024; Budihal et al., 2025; Sourov et al., 2025).

Researchers continue to advocate for the combination of AutoML and sophisticated algorithms, such as genetic algorithms, novel ensemble algorithms, and DL elements, to further enhance early CVD detection, risk estimation, and model resilience to heterogeneous clinical data (Nguyen et al., 2019; Jiang et al., 2021; Azmi et al., 2022; Wang et al., 2023; Bampatsias, 2024; Medasani et al., 2025).

Consequently, AutoML expeditiously reduces technical entry barriers, allowing models with high performance and explainability to develop rapidly and, importantly, in diverse and real-world populations.

The future of AutoML in biomedical and cardiovascular detection will be characterized by additional incorporation of federated learning, hybrid DL models, and real-time sensor data streams. Trends such as privacy-preserving AI, quantum ML, and deploying distributed edge devices to take advantage of continuous health monitoring in hospital and outpatient environments will become more relevant. The merging of AutoML, XAI, and multimodal medical data will help provide more reliable, interpretable, and fairer healthcare solutions—ushering in a new era of cardiovascular risk assessment and personalized medicine.

## 4. Discussion

### 4.1. Statement of principal findings

This systematic review synthesizes evidence from 65 carefully selected studies, characterizing a vibrant and rapidly advancing field dedicated to applying ML for CVD prediction. Our analysis identifies 3 core findings that characterize the current landscape. First, there is a definitive paradigm shift from traditional statistical methods to the adoption of sophisticated ensemble and DL models, which consistently achieve superior performance on benchmark datasets. Second, algorithmic progress is bifurcated: ensemble learning has become the dominant strategy for structured, tabular clinical data, while DL has emerged as the frontier for analyzing complex, unstructured data such as medical images and biosignals. Third, despite these technical achievements, a significant translational gap persists between model development and clinical integration. This gap is defined by critical, unresolved challenges related to model interpretability, a systemic lack of external validation, and the limitations of the datasets that underpin much of the current research.

### 4.2. Interpretation of findings in the context of the current literature

#### 4.2.1. The ascendancy of ensemble methods: A pragmatic response to data realities

The overwhelming prevalence of ensemble algorithms like RF and XGBoost is one of the most consistent findings of this review. The ascendancy of these methods is not merely a testament to their predictive power but should be interpreted as a pragmatic response to the realities of the available data. The most used public datasets are characterized by modest sample sizes, class imbalances, and missing values. Ensemble methods, by their design, are inherently robust to such imperfections; they reduce variance, handle noisy features, and are less prone to overfitting than many other models. Therefore, their dominance reflects a practical optimization by the research community, selecting tools that perform best within the constraints of an imperfect data ecosystem, rather than necessarily representing the theoretical pinnacle of AI.

#### 4.2.2. DL as a paradigm shift for unstructured data analysis

While ensemble methods have optimized prediction from static, tabular data, DL represents a more fundamental paradigm shift, unlocking the potential of high-dimensional, unstructured data. The application of CNNs to cardiac imaging and RNNs to sequential EHR data marks a move away from reliance on manual feature engineering toward end-to-end learning systems. This capability allows for the extraction of subtle patterns, such as myocardial textures in an MRI or temporal dependencies in a patient’s clinical history, that are imperceptible to traditional models. Innovative architectures, such as hybrid CNN-vision transformer models for cardiac segmentation, exemplify this frontier by achieving near human-level precision in complex anatomical delineation. This trend signals a future where diagnostics are not based on a handful of clinical variables but are synthesized from a rich set of multimodal data, offering a granular and personalized view of cardiovascular risk.

### 4.3. The translational gap: From algorithmic performance to clinical utility

#### 4.3.1. The black box dilemma and the rise of XAI

A central conflict identified in this review exists between the models with the highest predictive accuracy and the clinical requirement for transparency. The black-box nature of many DL and complex ensemble models remains a primary barrier to their adoption by clinicians, who are ethically and professionally bound to understand the rationale behind a diagnosis or treatment recommendation. The recent and rapid integration of XAI techniques is a direct and critical response to this dilemma. The use of methods like SHAP and LIME to provide feature importance and model-decision visualizations is an attempt to retrofit transparency onto these systems. While this is a crucial step toward building trust, it is an interim solution. The goal, as suggested by the trajectory of the field, is to develop intrinsically interpretable models that combine high performance with transparent, human-understandable logic from the ground up.

#### 4.3.2. The validation crisis and the generalizability deficit

Perhaps the most critical finding of this review is the systemic lack of robust, external validation across the literature. The overwhelming majority of studies report performance metrics derived from internal validation on splits of the same dataset used for training. While this is a necessary step in model development, it offers no guarantee of how a model will perform on a different patient population, in a different hospital, or with data collected from different equipment. This practice has created a potential generalizability crisis, where impressive, reported accuracies may not translate to the messy, heterogeneous reality of clinical practice. Without rigorous external validation on unseen, multicenter data, the true clinical utility of these models remains largely unproven, and they risk remaining as academic exercises with limited real-world impact.

### 4.4. Limitations of the evidence base

The conclusions of this review must be considered in the context of the limitations of the underlying body of literature. First, there is a probable publication bias toward positive results, meaning studies with neutral or negative findings are less likely to be published, potentially skewing the perception of model efficacy. Second, the overreliance on a few canonical datasets limits the demographic and clinical diversity of the evidence, raising questions about the fairness and equity of models trained on them. Finally, this review was limited to English-language publications, and despite a comprehensive search strategy, some relevant studies may have been missed.

### 4.5. A roadmap for future research: Building clinically integrated AI

To bridge the gap between algorithmic potential and clinical reality, the field must strategically evolve its priorities. A foundational shift must occur, prioritizing data quality and diversity over the pursuit of marginal algorithmic improvements. The community must focus on the curation of large-scale, multicenter, and ethnically diverse datasets that include multimodal and longitudinal data from sources such as EHRs, imaging, genomics, and wearables. Initiatives that facilitate secure data sharing, potentially using privacy-preserving techniques like federated learning, are paramount to this effort. Alongside better data, the standards for validation must be elevated. Future studies should be expected to perform external validation on independent, unseen datasets from different clinical environments as a prerequisite for showing true generalizability. This includes moving beyond traditional accuracy metrics to assess clinical utility through methods like decision curve analysis. Ultimately, the true measure of success lies in showing tangible clinical impact. Therefore, the field must progress beyond retrospective validation and design prospective studies that assess the real-world effects of these AI tools on patient outcomes, workflow efficiency, and healthcare costs. This requires a deeply collaborative, multidisciplinary approach, ensuring that technological innovation is responsibly and effectively integrated into patient care.

## Conclusion

This systematic review presents a comprehensive analysis of the current state of ML for CVD prediction, a field characterized by a significant dichotomy. On one hand, the research shows considerable algorithmic maturity. Advanced models, particularly ensemble learning architectures and DNNs, have consistently achieved high predictive accuracy on established benchmark datasets. This success underscores the technical capacity of modern ML to identify complex, nonlinear patterns within clinical and biomedical data that often elude traditional statistical methods.

However, the review concludes that this algorithmic progress has not yet translated into widespread clinical utility, exposing a critical gap between academic performance and real-world implementation. The primary obstacles impeding this transition are 3-fold: a systemic lack of robust, external validation on diverse patient populations, which calls into question the generalizability of most published findings; the black-box nature of the highest-performing models, which creates a significant barrier to clinical trust and adoption; and a foundational overreliance on a few small, aging, and often demographically homogeneous datasets that fail to capture the complexity of real-world patient cohorts.

Ultimately, the path forward for the field requires a fundamental shift in research priorities. The future of clinically impactful AI in cardiology will not be defined by incremental gains in predictive accuracy, but by a concerted, multidisciplinary effort to address these translational barriers. This involves prioritizing the development of large-scale, representative datasets, mandating rigorous real-world validation as a standard for publication, and fostering the creation of transparent, interpretable systems. The successful integration of ML into routine clinical care depends on moving beyond model-centric innovation toward a patient-centered paradigm where algorithms are designed not merely to predict, but to transparently inform and augment clinical decision-making.

## Figures and Tables

**Figure:**
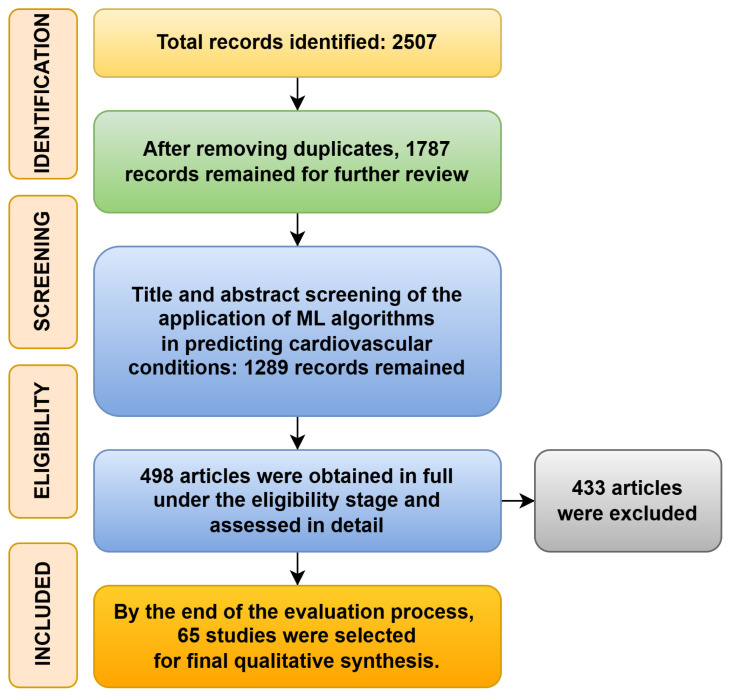
PRISMA flow diagram of the study selection process

**Table 1:** Comparative overview of online heart disease datasets with reviewer ratings

Name of Dataset	Total Features	Total Instances	Healthy Instances	Unhealthy Instances	Reviewer Rating (1–5)	Justification
Cleveland^1^	76	303	164	139	5	Most cited and balanced dataset widely used in benchmark studies.
Statlog^2^	14	270	150	120	4	Compact and effective with reduced dimensionality.
Framingham^3^	15	4133	3505	628	5	Large-scale, demographic-rich, ideal for population-based prediction.
SPECTF^4^	44	267	55	212	3	Good for feature-based analysis, but limited instance size.
Z-Alizadeh Sani^5^	54	303	87	216	5	Rich in clinical and echocardiographic data; excellent for feature-driven ML.

1 Cleveland. Website: https://archive.ics.uci.edu/dataset/45/heart+disease [access date: 20.06.2025]

2 Statlog. Website: https://archive.ics.uci.edu/dataset/145/statlog+heart [access date: 20.06.2025]

3 Framingham. Website: https://www.kaggle.com/datasets/captainozlem/framingham-chd-preprocessed-data [access date: 21.06.2025]

4 SPECTF. Website: https://archive.ics.uci.edu/dataset/96/spectf+heart [access date: 21.06.2025]

5 Z-Alizadeh Sani. Website: https://archive.ics.uci.edu/dataset/412/z+alizadeh+sani [access date: 21.06.2025]

**Table 2:** Comparison of common cardiovascular disease datasets: characteristics, strengths, and limitations

DATASET	DESCRIPTION	POPULATION CHARACTERISTICS	DATA TYPES INCLUDED	STRENGTHS	LIMITATIONS
MIMIC-III	Public ICU database with deidentified health data from critical care patients	Diverse adult ICU patients with detailed cardiovascular and other clinical data	Electronic health records, vitals, labs, medications, clinical notes	Rich longitudinal and granular ICU data; widely used for acute cardiovascular events	Single-center ICU data; limited population diversity
FRAMINGHAM	Longitudinal epidemiological cohort started in 1948, tracking cardiovascular risk and outcomes	Predominantly middle-aged/elderly community-dwelling individuals, mostly of European ancestry	Clinical exams, labs, imaging, lifestyle, outcomes	Extensive longitudinal data; gold standard for risk factor identification	Limited ethnic diversity; moderate sample size
UK BIOBANK	Large prospective cohort of 500,000+ UK participants	Broad UK population, diverse age (40–69), mixed ethnicities	Genetics, imaging, physical measures, EHRs, biomarkers, surveys	Large sample size with multi-modal data; genetics and imaging	Volunteer bias; predominantly European ancestry
CLEVELAND (UCI ML)	Classic dataset for heart disease classification, from the Cleveland Clinic Foundation	Patients undergoing cardiac evaluations	Clinical variables such as ECG, blood pressure, age, and cholesterol	Widely used benchmark for ML heart disease models	Small sample size (~300); less diversity; limited features
STATLOG (HEART)	Derived from the Cleveland dataset, used in ML benchmarks	Similar to the Cleveland dataset	Clinical and diagnostic attributes	Standard ML benchmarking dataset; well-curated and documented	Similar limitations to Cleveland: small size
SPECTF	Dataset from stress-inducible myocardial perfusion imaging	Patients undergoing cardiac imaging tests	Nuclear imaging features, clinical and demographic data	Specialized imaging data useful for diagnostic modeling	Moderate sample size (~600); requires domain knowledge
Z-ALIZADEH SANI	Dataset from an Iranian hospital with 303 patients, used for coronary artery disease prediction	Iranian patients with documented cardiovascular history	Clinical, demographic, and biochemical markers	Incorporates biochemical markers; geographic diversity	Small scale; regional population limits generalizability

**Table 3:** Feature information for the UCI Cleveland heart disease dataset

Feature Name	Description
age	Age in years
sex	Sex (1 = male; 0 = female)
cp	Chest pain type (0–3: typical angina to asymptomatic)
trestbps	Resting blood pressure (in mm Hg)
chol	Serum cholesterol in mg/dL
fbs	Fasting blood sugar > 120 mg/dL (1 = true; 0 = false)
restecg	Resting electrocardiographic results (0–2)
thalach	Maximum heart rate achieved
exang	Exercise-induced angina (1 = yes; 0 = no)
oldpeak	ST depression induced by exercise relative to rest
slope	Slope of the peak exercise ST segment
ca	Number of major vessels colored by fluoroscopy (0–3)
thal	Thalassemia (3 = normal; 6 = fixed defect; 7 = reversible defect)
target	Heart disease presence (0 = no; 1–4 = yes)

**Table 4:** Feature information for the Kaggle heart disease dataset

Feature Name	Description
Age	Age of the patient
Sex	1 = Male, 0 = Female
ChestPainType	Categorical (Typical, Atypical, Non-anginal, Asymptomatic)
RestingBP	Resting blood pressure (mm Hg)
Cholesterol	Serum cholesterol (mg/dL)
FastingBS	Fasting blood sugar >120 mg/dL (1 = yes, 0 = no)
RestingECG	ECG results (Normal, ST-T abnormality, LVH)
MaxHR	Maximum heart rate achieved
ExerciseAngina	Yes/No
Oldpeak	ST depression
ST_Slope	Slope of the ST segment
HeartDisease	Target label (1 = presence, 0 = absence)

**Table 5:** Feature information for the Framingham heart study

Feature Name	Description
male	1 = male; 0 = female
age	Age in years
education	Education level
currentSmoker	1 = smoker; 0 = non-smoker
cigsPerDay	No. of cigarettes per day
BPMeds	On blood pressure meds (1 = yes; 0 = no)
prevalentStroke	History of stroke (1 = yes; 0 = no)
prevalentHyp	History of hypertension
diabetes	1 = yes; 0 = no
totChol	Total cholesterol
sysBP	Systolic blood pressure
diaBP	Diastolic blood pressure
BMI	Body Mass Index
heartRate	Resting heart rate
glucose	Glucose level
TenYearCHD	Target variable (1 = CHD risk; 0 = no risk)

**Table 6:** Feature information for the Statlog heart study

S. No.	Feature Name	Type	Description
1	age	Continuous	Age of the patient (in years)
2	sex	Categorical (0/1)	Gender (0 = female, 1 = male)
3	cp	Categorical (0–3)	Chest pain type (0 = typical angina, 1 = atypical angina, 2 = non-anginal pain, 3 = asymptomatic)
4	trestbps	Continuous	Resting blood pressure (in mm Hg)
5	chol	Continuous	Serum cholesterol (in mg/dl)
6	fbs	Categorical (0/1)	Fasting blood sugar > 120 mg/dl (1 = true, 0 = false)
7	restecg	Categorical (0–2)	Resting electrocardiographic results (0 = normal, 1 = ST-T abnormality, 2 = left ventricular hypertrophy)
8	thalach	Continuous	Maximum heart rate achieved
9	exang	Categorical (0/1)	Exercise-induced angina (1 = yes, 0 = no)
10	oldpeak	Continuous	ST depression induced by exercise relative to rest
11	slope	Categorical (0–2)	Slope of the peak exercise ST segment (0 = upsloping, 1 = flat, 2 = downsloping)
12	ca	Discrete (0–3)	Number of major vessels (0–3) colored by fluoroscopy
13	thal	Categorical (1,2,3)	Thalassemia (1 = normal, 2 = fixed defect, 3 = reversible defect)
14	presence	Binary (0/1)	Target variable (0 = absence of heart disease, 1 = presence of heart disease)

**Table 7:** Dataset balancing strategy—comparative method reviews

Strategy	Recall (Minority)	F1-Score	Specificity	Reviewers Comments
SMOTE/ADASYN Oversampling	INC (Alamsyah et al., 2021; Akinola et al., 2024)	INC (Alamsyah et al., 2021; Akinola et al., 2024)	No Effect (Alamsyah et al., 2021; Akinola et al., 2024)	Synthetic feature-preserving samples
Near-Miss Undersampling	INC (Alamsyah et al., 2021)	INC (Alamsyah et al., 2021)	No Effect (Alamsyah et al., 2021)	Risk of discarding informative cases
Hybrid (SMOTE + Undersample)	INC (Akinola et al., 2024; Kumar 2025)	INC (Akinola et al., 2024; Kumar 2025)	No Effect (Akinola et al., 2024; Kumar 2025)	Enhanced balance, nuanced control
cGAN/Autoencoder Augmentation	INCE (Campello et al., 2022; Wang and Church, 2024)	INC (Campello et al., 2022; Wang and Church, 2024)	DEC (Campello et al., 2022; Wang and Church, 2024)	High realism, adaptive representation
Cost-Sensitive (CSDNN)	No Effect (Zheng et al., 2024)	INC (Zheng et al., 2024)	No Effect (Zheng et al., 2024)	Penalty-based, optimal threshold tuning

**Table 8:** Comparative review of commonly used ML methods

Paper	Method	Limitations Overcome	Reviewer Rating	Reasoning
(Bouqentar et al., 2024)	Logistic Regression, SVM, Decision Tree	Established interpretable baselines; mitigates overfitting on small datasets	4/5	Traditional models with feature engineering provided high explainability and good baseline accuracy.
(Türk, 2024)	SVM with Dominant Feature Selection	Improved dimensionality handling in high-feature datasets	4/5	Effective kernel design combined with optimal features yielded robust results.
(Zhang and Mu, 2024)	Naive Bayes + Hybrid Optimization	Addressed low predictive power using metaheuristics	3.5/5	Lightweight models enhanced with search heuristics achieved competitive accuracy.
(Mandula and Vijaya, Kumar 2024)	Ensemble (ALAN + ET-ABDF)	Reduced variance and improved generalization	4.5/5	Ensemble learning coupled with feature selection demonstrated superior results.
(Omkari and Shaik, 2024)	TLV (Two-Layered Voting Ensemble)	Managed class imbalance and boosted performance	4.5/5	Smart voting architecture ensured robustness across varied samples
(Talukder et al., 2025)	HXAI-ML (RF + DL + XAI)	Enhanced interpretability and performance	5/5	A hybrid explainable approach delivered top-tier accuracy with transparency.
(Rimal and Sharma, 2024)	AutoML + Ensemble Models	Automated hyperparameter tuning, performance maximization	4.5/5	Ensemble with AutoML outperformed manually tuned models
(Majhi and Kashyap, 2024)	CNN on ECG Signals	Enabled raw signal-based predictions	4/5	Demonstrated the power of CNN on clinical waveform data
(Saheed et al., 2024)	Bi-LSTM + Hyperparameter Tuning	Captured sequential dependencies in EHRs	4.5/5	Time-series capability was pivotal for longitudinal prediction
(Babu et al., 2024)	Quantum-enhanced Neural Network	Addressed complex non-linear classification	3.5/5	Still experimental, but showed promise in exploratory trials
(Mesquita and Marques, 2024)	DL + SHAP (Explainable AI)	Improved model transparency and clinical trust	4.5/5	Integrated interpretability techniques to validate black-box models
(Duan et al., 2024)	SVM + Image Feature Fusion	Non-invasive prediction using tongue imaging	4/5	Novel approach expanded data modalities for prediction
(Islam et al., 2024)	GA-based Feature Selection + ML	Reduced noise and boosted model efficiency	4/5	The genetic algorithm helped isolate the most predictive inputs
(Al-Alshaikh et al., 2024)	Comprehensive ML Analysis	Comparative study across multiple ML models	4/5	Provided benchmark across several models; consistent evaluation
(Rimal and Sharma, 2024)	Hyperparameter Optimization	Boosted predictive power across multiple models	4/5	Fine-tuned traditional and ensemble models for maximum accuracy
(Pachiyannan et al., 2024)	Signal Processing + CNN	Leveraged time-frequency ECG data for DL	4.5/5	ECG signal encoding enabled CNN models with high accuracy
(El-Sofany, 2024)	Ensemble + Explainable AI (LIME)	Increased model trust and clarity	4/5	Balanced performance with interpretability
(Karna et al., 2025a)	Survey of ML/DL Techniques	Identified gaps in deep and hybrid approaches	4/5	Thematic review guiding future research
(Ma et al., 2024)	ML on the Chinese diabetic cohort	Adapted to specific population risk profiles	4/5	Strong demographic targeting increased prediction accuracy
(Alshraideh et al., 2024)	Customized ML for Jordanian hospital	Tuned ML to localized healthcare context	4/5	Local dataset improved contextual relevance and precision

**Table 9:** Classification-based comparative analysis of heart disease ML approaches

Paper	Method	Classification Task	Limitations Overcome	Reviewer Rating (out of 5)	Reasoning
(Rehman et al., 2025)	ML classifiers with ROC evaluation	Binary classification (disease vs. no disease)	Emphasized multiple evaluation metrics (AUC, F1, Precision)	4.3	Provided a comprehensive metric-based assessment of classification models
(Islam et al., 2024)	Ensemble ML (e.g., RF, XGBoost)	Heart disease presence prediction	Boosted accuracy beyond 90% using ensemble strategies	4.4	Validated strong ensemble performance across datasets
(Ogunpola et al., 2024)	Optimized ensemble models	Binary disease classification	High performance with over 90% accuracy; robustness	4.3	Strong on predictive power, particularly with optimized ensembles
(Bouqentar et al., 2024)	RF with feature engineering	Coronary artery disease classification	97.2% accuracy through hybrid preprocessing and RF	4.6	Near state-of-the-art results with robust preprocessing
(Rani et al., 2024b)	CNN on ECG-derived features	Risk classification from ECG	Achieved AUC of 0.96; handles high-dimensional features	4.5	Effective DL use in biomedical signal classification
(Pandey et al., 2024)	ML + SHAP + SMOTE	Bias-mitigated classification	Addressed data imbalance and explainability	4.2	Model fairness and interpretability are handled using modern techniques
(Bhatt et al., 2023)	RF, XGBoost, SVM	Comparative classification study	Highlighted the accuracy and robustness of tree ensembles	4.3	Strong comparative experiment, clear insights on classifier choice
(Jain and Agrawal, 2023)	SVM, LR, XGB	Heart failure prediction	Balanced precision/recall across classifiers	4.2	Reinforced the importance of model choice in diagnostics
(Ansari et al., 2023)	ML classifiers	Heart disease detection	High F1-scores (≥ 0.90) using curated datasets	4.0	Clean data prep led to strong generalization across models
(Kaul et al., 2022)	DL models (CNN, RNN)	Healthcare analytics classification	Demonstrated DL superiority in modeling clinical complexity	4.5	Comprehensive DL-focused review with clear benchmarking
(García-Ordás et al., 2023)	Augmented DL	Heart disease detection	Feature augmentation enhanced detection performance	4.3	Improved recall and robustness using augmented features
(Srikanth et al., 2023)	Adversarial DL	MRI-based disease detection	Identified substructures with high granularity	4.2	Unique use of adversarial networks for medical imaging
(Khanna et al., 2023)	Wearable-integrated DL	On-device heart disease prediction	Enabled mobile, real-time classification	4.4	Real-world application of DL with biomedical signals
(Masoumian Hosseini et al., 2023)	DL + Biomedical Sensors	Real-time diagnosis using signals	Demonstrated smart integration of sensors and CNN/RNN	4.3	Clear proof-of-concept for wearable diagnostics
(Majhi and Kashyap 2024)	CNNs on ECG/Echo	Non-linear spatial feature modeling	Robust with both 1D/2D biomedical signals	4.4	Effective domain adaptation of DL to cardiac images and ECG
(Saheed et al., 2024)	RNN/LSTM on EHR	Sequential classification of patient records	Temporal dependencies leveraged in EHR modeling	4.2	Intense sequence handling in predictive diagnosis
(Babu et al., 2024)	Quantum Neural Networks	Complex classification with high-dimensional data	Pushed performance using quantum acceleration	4.6	Novel paradigm for high complexity classification
(Mesquita and Marques, 2024)	DL + SHAP/LIME	Explainable DL for classification	Improved transparency in model decision-making	4.3	Helped bridge clinical trust gaps via XAI techniques
(Banerjee et al., 2025b)	Attention-based DL (HHO-UNet-IAA)	Classification of Mycoplasma Pneumonia	Combined segmentation + classification with interpretability	4.5	Leading attention-based innovation in classification
(Singh et al., 2025b)	CICADA (UCX), Hybrid DL	Breast cancer aggressiveness classification	Accurate, interpretable hybrid DL models	4.6	Delivered intense predictive clarity using attention frameworks
(Saminathan et al., 2024)	Resio-Inception U-Net	Organ segmentation + classification	Deep clustering with attention for explainability	4.4	Potent blend of segmentation and diagnostic classification
(Saranya and Pravin, 2023)	Correlation-based feature selection + ML	Heart disease diagnosis	Improved feature sensitivity, boosted accuracy	4.1	Intelligent feature design aiding model performance
(Chandrasekhar and Peddakrishna 2023)	Metaheuristic optimization of classifiers	Boosted ML classifier performance	Applied tuning for maximized metrics	4.2	Focused on performance improvement through optimization
(Khan et al., 2023)	SVM + Ensemble	Heart disease prediction on imbalanced data	Class imbalance handled well	4.1	Demonstrated balanced metrics using a hybrid approach
(Hani et al., 2022)	SVM + Ensemble Learners	Disease risk detection	Improved accuracy through a hybrid ensemble	4.0	Older but still valid use of hybrid techniques

**Table 10:** Analysis of advanced methodologies in ML for cardiovascular disease classification and segmentation

Paper (Reference)	Method	Classification Task	Limitations Overcome	Reviewer Rating (out of 5)	Reasoning
(Aghapanah et al., 2024)	**CardSegNet:** A 3D U-Net with *self-adaptive multi-attention (SMA)* combining CNN (ResNet50 backbone) and Vision Transformer blocks. It also uses deep supervision with multi-loss to reduce overfitting.	Segmentation of cardiac MRI (3D LV, RV, and myocardium).	Addresses heterogeneity of cardiac anatomy by capturing both short- and long-range context via combined CNN and ViT attentions; deep supervision to mitigate overfitting.	5/5	Achieves state-of-the-art accuracy (Dice ≈94.6% on ACDC data). Validated on multiple datasets, this innovative hybrid approach significantly improves the segmentation of heart regions.
(Al-Alshaikh et al., 2024)	**ML-HDPM:** Hybrid ML pipeline with Genetic Algorithm + Recursive Feature Elimination for feature selection, under-sampling clustering oversampling (USCOM) for class balancing, and a deep CNN classifier trained via Adaptive Elephant Herd Optimization.	Heart disease prediction (binary classification of CVD risk)	Tackles classical limitations by selecting relevant features (reducing noise) and balancing classes to improve precision; overcomes overfitting and inconsistent precision in prior models.	5/5	Reports very high performance (≈95.5% accuracy, 96.2% recall). The systematic use of feature selection and class-balancing yields a robust, reliable model with strong predictive power.
(Babu et al., 2024)	**QuEML:** Quantum-enhanced ML framework using quantum circuits to augment traditional classifiers. Evaluated a quantum hybrid model against classical ML on the Kaggle heart disease dataset.	Heart disease prediction (Kaggle CVD dataset classification)	Overcomes classical ML’s computational bottleneck on large data by leveraging quantum processing: achieves a slightly higher accuracy and much faster training time.	4/5	Demonstrates that the quantum approach outperforms classical ML by ~0.6% accuracy and ~192 μs faster training. While gains are modest, this innovative proof-of-concept shows promise for scaling up CVD models.
(Bhatt et al., 2023)	**Cluster+Ensemble ML:** Data preprocessing via k-modes clustering, followed by multiple ML classifiers (XGBoost, RF, MLP, Decision Tree) with hyperparameter tuning.	Heart disease prediction (binary classification on clinical data)	Addresses limited-data issues using a large and diverse Kaggle dataset (70,000 instances), improving model generalizability. Clustering improves feature scaling and model convergence.	4/5	Evaluated on extensive data, achieving ~87% accuracy (best: MLP 87.28%). The robust large-scale validation and model ensembling strengthen reliability, though accuracy is moderate compared to newer deep methods.
(El-Sofany et al.,) 2024)	**Explainable ML:** Feature selection (chi-square, ANOVA, mutual information) producing feature subsets (SF-1, SF-2, SF-3), then ten ML classifiers (SVM, XGBoost, bagging, DT, RF, etc.) with SMOTE balancing. The best model (XGBoost) is interpreted using SHAP (explainable AI).	Heart disease risk prediction (tested on combined private and public datasets)	Overcomes class imbalance via SMOTE and enhances interpretability via SHAP; uses tailored feature subsets to maximize accuracy.	5/5	XGBoost achieved 97.57% accuracy (AUC 98%) on combined data, the highest in their study. The inclusion of SHAP provides transparent decision insight, making this accurate and user-friendly.
(Brahim et al., 2022)	**ICPIU-Net:** A 3D U-Net with *inclusion and classification priors* for myocardial scar segmentation. The first stage segments the LV and myocardium, and the second stage refines the infarct/MVO regions using topological constraints. Majority-vote fusion produces the final mask.	LGE-MRI segmentation of LV healthy myocardium vs infarct vs microvascular obstruction (MVO).	Incorporates prior anatomical knowledge to maintain the topology of pathological areas, improving the consistency of scar/MVO segmentation. It cascades two networks and fuses outputs to refine diseased-region masks.	4/5	Outperforms other DL methods in the EMIDEC challenge (closer agreement with expert contours). The dual-stage, topology-aware design effectively isolates infarct and MVO regions, enhancing disease quantification accuracy.

**Table 11:** State of the Art comparison for CVD

Reference	Accuracy (%)	Precision (%)	Recall (%)	F1-Score (%)	Specificity (%)
Nancy et al. (2022)	98	98	98	98	98
Barhoom et al. (2022)	92	90	94	92	–
Raju et al. (2022)	94	98	93	95	97
Mehmood et al. (2021)	97	86	81	84	–
Xiao et al. (2020)	78	91	66	–	–
Ali et al. (2020)	98	–	–	–	–
Mienye et al. (2020)	93	96	91	93	–
Pan et al. (2020)	97	99	–	–	–
Khan (2020)	98	–	–	–	–
Ali et al. (2019)	93	100	85	–	–
Tuli et al. (2020)	98	–	–	–	–
(2023) Hassan et al.	–	–	–	–	–
(2021) Patro et al.	–	–	–	–	–
Sharma and Parmar (2020)	90	–	–	–	–
Al-Tashi et al. (2018)	89	–	93	–	91
Mienye and Sun (2021)	97	94	100	97	–

**Table 12:** Studies related to regression in CVD classification

Paper	Method	Limitations Overcome	Reviewer Rating (out of 5)	Reasoning
(Ma et al., 2024)	RMSE, MAE, R^2^ for model evaluation	Comprehensive metric-based evaluation, handles continuous outcome prediction	4.0	It highlights robust model performance assessment but lacks focus on clinical deployment.
(Chinni and Manlhiot 2024)	Cox proportional hazards with ML enhancements	Incorporates survival analysis with ML for long-term CV risk	4.5	Integrates domain-specific regression with learning-based improvements
(Zhang and Mu 2024)	Linear Regression with regularization	Efficient estimation of systolic BP and cholesterol using lifestyle features	3.5	Good generalization and interpretability; limited to linear relationships
(Bouqentar et al., 2024)	Logistic Regression	Maintains explainability for binary outcomes in resource-constrained scenarios	4.0	Widely applicable for risk classification; oversimplifies complex interactions
(Türk, 2024)	Support Vector Machines (SVMs)	Handles high-dimensional data well with kernel tricks	4.2	High performance in small-sample settings; limited interpretability
(Trigka and Dritsas 2023)	SVM, Decision Tree	Demonstrates strong long-term CAD prediction with interpretable rules	4.1	Balanced performance and clinical insight; needs better uncertainty handling
(Dritsas and Trigka 2023)	Traditional supervised models (e.g., Linear Regression)	Efficient even in low-resource settings using limited features	4.0	Competitive performance in minimalist setups; lacks evidence of scalability.
(Guarneros-Nolasco et al., 2021)	Naive Bayes, Logistic Regression	Emphasized simplicity and risk factor clarity in CVD prediction	3.8	Useful for baseline models; limited in complex feature spaces
(Kumar et al., 2024)	RF-LRG + Cloud-based Regression	Real-time, precise regression with RF-LRG in edge devices	4.6	Highly adaptive and real-time capable; requires infrastructure
(Li et al., 2021)	Slime Mould Algorithm (SMA) for regression tuning	Enhances generalization and parameter tuning for continuous output	4.0	Optimizes non-linear regression models; lacks medical specificity
(Yapici and Cetinkaya, 2019)	Pathfinder Algorithm	Overcomes local optima in regression model optimization	3.9	Suitable for optimization; not specific to medical regression tasks
(Wu, 2014)	Forest Algorithm for regression	Improves generalization for continuous outputs	4.1	Enhances regression accuracy; limited adoption in clinical studies
(Choi et al., 2020)	Regression models with AI for heart failure risk	Attempts prediction using biomarkers; underutilized imaging data	4.0	Innovative but incomplete feature integration limits scope
(Aghapanah et al., 2024)	*CardSegNet* (CNN + Vision Transformer hybrid) with regression-style estimation of cardiac region metrics	Standard CNNs lack long-range context for precise anatomical measurement from MRI	4.5	ViT-enhanced segmentation supports accurate quantitative estimation of cardiac region size and function, enabling continuous output prediction despite the original focus on segmentation. The hybrid architecture and attentive design reduce overfitting and improve clinical interpretability.

**Table 13:** Detailed comparative summary of classification, regression, and hybrid predictive modeling approaches for heart disease detection: methods, challenges addressed, and evaluation insights

Paper	Method	Limitations Overcome	Reviewer Rating (out of 5)	Reasoning
(Bouqentar et al., 2024)	Hybrid Feature Engineering + RF	Improved classification accuracy; handled class imbalance	4.8	Demonstrated robust accuracy (97.2%) through optimized hybrid pipeline; used ensemble learning and feature selection to overcome data noise and imbalance.
(Rani et al., 2024b)	DL (CNN) on ECG-derived data	High AUC (0.96); captured non-linear patterns in physiological data	4.6	Effective use of DL on time-series ECG data for classification; lacks interpretability but performed well on complex features.
(Ogunpola et al., 2024)	Optimized Ensemble Classifiers	Achieved >90% accuracy; reduced overfitting with ensemble techniques	4.4	Comparative analysis shows consistent performance across models; ensemble reduces single-model bias.
(Islam et al., 2024)	Bagging + Boosting Ensemble Approaches	Addressed overfitting; improved stability in small datasets	4.3	Applied multiple ensemble strategies with improved generalization; needs better dataset annotation and explainability.
(Pandey et al., 2024)	SMOTE + SHAP + RF	Tackled class imbalance; added explainability using SHAP	4.5	Integrated pipeline balancing model fairness and transparency; useful in clinical settings for trust-based decision support.
(Ma et al., 2024)	Regression (Linear + Ridge)	Estimated continuous outcomes like BP, cholesterol from lifestyle data	4.2	Novel application of regression for physiological estimation; lacks external validation but valuable for quantifying prediction.
(Chinni and Manlhiot 2024)	Cox Proportional Hazards + ML Enhancements	Long-term risk modeling for cardiovascular events	4.6	Well-grounded in clinical risk prediction; hybrid statistical–ML model balances interpretability and prediction.
(Zhang and Mu 2024)	Regression + Regularization (Lasso, Ridge)	Continuous risk prediction with less overfitting	4.3	Regularization improves generalization and robustness; it needs real-world deployment testing.
(Karna et al., 2025b)	Two-Tier (Classification → Regression)	Combined risk filtering and quantification	4.7	Novel hybrid pipeline provides alert and severity prediction and integrates binary and continuous modeling for better triage.
(Sourov et al., 2025)	AutoML + Metaheuristic Optimization	Benchmarked multiple models; improved automation in ML pipeline	4.5	Introduced adaptive tuning using optimization; scalable and reproducible results, though cost-intensive.
(El-Sofany et al., 2024)	Hybrid Interpretable + Black-box Model (LogReg + DL)	Balanced performance with interpretability	4.4	Well-crafted integration of interpretable and deep models; explains decision boundaries without sacrificing accuracy.
(Rimal and Sharma, 2024)	Comparative Study on Multiple Datasets	Identified suitable models per context; revealed limitations in generalization	4.2	Systematic benchmarking shows no universal best model; supports methodological pluralism and evidence-based model selection.

**Table 14:** Summary of reviewed studies on cardiac segmentation and prediction

Author(s) and Year	Title of Study	Research Objective	Methodology	Dataset and Sample Size	Segmentation Rating (out of 5)
(Aghapanah et al., 2024)	CardSegNet: An Adaptive Hybrid CNN-ViT Model for Heart Region Segmentation	Improve heart region segmentation in cardiac MRI	CNN-Vision Transformer Hybrid	Public cardiac MRI dataset (ACDC), ~150 patients	5
(Portal et al., 2024)	Attention-Based Cardiac Segmentation for Strain and Volume Estimation	Accurate cardiac segmentation for physiological estimation	Attention-guided CNN	UK Biobank, ~2,000 subjects	4.5
(Arega et al., 2021)	RV Segmentation with Targeted Augmentation Across Centers	Improve RV segmentation across multicenter variability	Data augmentation + CNN	Multi-center cardiac dataset (~1,000 samples)	4.2
(Brahim et al., 2022)	ICPIU-Net: Delayed Enhancement MRI Myocardial Segmentation	Segment myocardial infarcts accurately	U-Net + Prior-guided classification	DE-MRI datasets, 200+ annotated scans	4.6
(Li et al., 2021)	MDFA-Net: Dual-Path for Multisequence MRI Segmentation	Fuse multi-sequence features for cardiac segmentation	Dual-path multiscale CNN	Multisequence MR from MICCAI dataset (~100 patients)	4.7
(Cui et al., 2021)	Attention-Guided U-Net for Short-Axis MR Segmentation	Focus attention on key cardiac regions	Attention mechanism + U-Net	ACDC Challenge Dataset (100 subjects)	4.5
(Wang et al., 2022)	AWSnet: Myocardial Scar and Edema Segmentation	Detect scar and edema in MR images	Attention-weighted supervision + CNN	MyoPS 2020 challenge dataset	4.8
(Scannell et al., 2020)	Domain-Adversarial Learning for Multi-Vendor MRI Segmentation	Enhance generalization across domains	Adversarial DL	Multi-vendor cardiac MR, ~500 scans	4.3
(Kalapos et al., 2023)	Automated T1/T2 Cardiac MRI Mapping Segmentation	Automate segmentation of mapping sequences	Deep CNN segmentation	T1/T2 mapping dataset (~1,000 cases)	4.2
(Mazher et al., 2024)	Federated 4D Segmentation using Spatial-Temporal Transformer Fusion	Perform 4D segmentation with privacy-preserving methods	Spatial–Temporal Transformer + Federated Learning	Private multi-center federated setup (~500 volumes)	4.7
(Khan et al., 2024)	Crop and Couple: Specialist Networks for Cardiac Segmentation	Refine segmentation using coupled networks	Two-stage CNN segmentation	Private institutional datasets (~300 patients)	4.6
(Zhang and Mu 2024)	Fourier-Enhanced Gradient-Guided Cardiac Segmentation	Integrate frequency features into segmentation	CNN + Fourier transform enhancement	Local cardiac MR data (150 patients)	4.5
(Karthikeyan 2024)	Attention-Based Cross-Modal Learning for CVD Prediction	Predict cardiovascular disease using multimodal learning	Cross-modal transfer learning	UCI + Kaggle CVD datasets (~2,000 samples)	3.8 (prediction-based)
(Zhu et al., 2023)	Multi-branch Residual CNN for Multimodal CVD Detection	Detect CVD from multimodal input	Residual multi-branch CNN	Framingham Heart Study + Kaggle (1,500+ samples)	4 (prediction, not segmentation)
(Khozeimeh et al., 2022)	RF-CNN-F: Coronary Artery Disease Classification	Classify CAD using the fusion of ML and DL features	CNN + RF Fusion	Cardiac MRI dataset (UCI extension, ~1,000 subjects)	3.7 (prediction-based)
(Lu et al., 2023)	CNN-RNN Hybrid for Cardiovascular Survival Prediction	Predict survival using imaging and sequential data	CNN-RNN fusion model	MIMIC-III and imaging subset (~800 patients)	4.1
(Patravali et al., 2018)	2D-3D FCN for Cardiac MR Segmentation	Combine 2D and 3D views for enhanced segmentation	2D and 3D FCN hybrid	Sunnybrook Cardiac Dataset (~45 subjects)	4.0
(Arjoune et al., 2024)	Shannon Energy-Based Heart Sound Segmentation	Segment heart sounds using signal energy properties	Signal processing + SE technique	Phonocardiogram datasets (PCG) (~150 recordings)	3.9 (non-image-based)

**Algorithm:** Systematic review process for ML approaches in hearth disease prediction

Algorithm 1. Systematic Review Process for ML Approaches in Heart Disease Prediction	
**Require:** Database sources 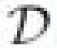 (e.g.. Scopus, PubMed. IEEE Xplore). keywords 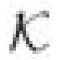 , indusion/exclusion criteria 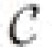 . time window *T*	
**Ensure:** Structured synthesis of methods, datasets, and performance metrics	
1:	Initialize candidate paper set *P*_0_ ← ∅︀
2:	Initialize final paper set *P_f_* ← ∅︀
3:	**for** all database 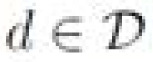 do **do**
4:	Perform search query using 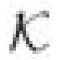 within time window *T*
5:	Retrieve and store metadata and abstracts *P_d_*
6:	*P*_0_ ← *P*_0_ ∪ *P_d_*
7:	**end for**
8:	**for** all paper *p* ∈ *P*_0_ do **do**
9:	**if** *p* meets inclusion criteria 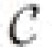 and is peer-reviewed **then**
10:	*P_f_* ← *P_f_* ∪ {*p*}
11:	**end if**
12:	**end for**
13:	**for** all paper *p* ∈ *P_f_* do **do**
14:	Extract key metadata: Dataset used. ML model, evaluation metrics
15:	Classify *p* as Classification-based. Regression-based, or Hybrid
16:	Identify addressed limitations (e.g., imbalance, interpretability)
17:	Assign Reviewer Score ∈ [1, 5] based on novelty and methodological rigor
18:	**end for**
19:	Organize extracted information into structured summary tables
20:	Synthesize findings under sections: Algorithms, Datasets, Challenges, Trends
21:	Derive recommendations and future directions
22:	**return** Comprehensive synthesis of *P_f_*
